# Exploring Cyclodextrin-Based Nanosponges as Drug Delivery Systems: Understanding the Physicochemical Factors Influencing Drug Loading and Release Kinetics

**DOI:** 10.3390/ijms25063527

**Published:** 2024-03-20

**Authors:** Bartłomiej Pyrak, Karolina Rogacka-Pyrak, Tomasz Gubica, Łukasz Szeleszczuk

**Affiliations:** 1 Doctoral School, Medical University of Warsaw, Żwirki i Wigury 81, 02-093 Warsaw, Poland; bartlomiej.pyrak@wum.edu.pl; 2 Department of Organic and Physical Chemistry, Faculty of Pharmacy, Medical University of Warsaw, Banacha 1, 02-097 Warsaw, Poland; lukasz.szeleszczuk@wum.edu.pl; 3 Central Clinical Hospital of the Ministry of Interior and Administration, Wołoska 137, 02-507 Warsaw, Poland; karolinarogacka2697@gmail.com

**Keywords:** cyclodextrin, nanosponge, drug delivery system, synthesis, physicochemical

## Abstract

Cyclodextrin-based nanosponges (CDNSs) are complex macromolecular structures composed of individual cyclodextrins (CDs) and nanochannels created between cross-linked CD units and cross-linkers. Due to their unique structural and physicochemical properties, CDNSs can possess even more beneficial pharmaceutical features than single CDs. In this comprehensive review, various aspects related to CDNSs are summarized. Particular attention was paid to overviewing structural properties, methods of synthesis, and physicochemical analysis of CDNSs using various analytical methods, such as DLS, PXRD, TGA, DSC, FT-IR, NMR, and phase solubility studies. Also, due to the significant role of CDNSs in pharmaceutical research and industry, aspects such as drug loading, drug release studies, and kinetics profile evaluation of drug–CDNS complexes were carefully reviewed. The aim of this paper is to find the relationships between the physicochemical features and to identify crucial characteristics that are influential for using CDNSs as convenient drug delivery systems.

## 1. Introduction

The European Medicines Agency (EMA) distinguishes almost 600 different types of dosage forms in standard use, a large share of which are oral medications [1]. According to financial reports, oral formulations contributed to about 42.2% of the drug market share in 2021, of which 53.1% was held by tablets alone [2,3]. Pharmaceuticals prepared for oral intake are convenient to use, but their activity is disturbed due to diverse processes of drug disposition in the organism—absorption, distribution, metabolism, and excretion (ADME). Since the efficiency of the drugs is dose-dependent, absorption is usually named as the most influential part of the metabolic journey of orally administered pharmaceuticals. In the case of extremely poor oral intake characteristics, e.g., instability in gastrointestinal environment or intestinal wall permeability limitations due to the structural properties or electric charge of the drug, a new route of administration should be considered. No drug presents perfect pharmacokinetic parameters in oral use; thus, the formulation development in the pharmaceutical industry is still valid.

Knowing that the drug displays a suitable pharmacodynamic effect, research on improving bioavailability and reducing adverse effects is required. These objectives can be achieved by chemical modification of the drug itself (e.g., the formation of prodrugs or better-absorbed drug forms) or by implementation of a pharmaceutical into the transport systems. Finding the optimal drug delivery system for a given drug is difficult, due to a series of factors, like complex stability, the toxicity of the formulation, or the influence on the drug’s pharmacokinetic parameters or synthesis costs, which are crucial for proper therapeutic effectiveness and optimization of the technological processes of drug formulations’ production.

**Figure 1:**
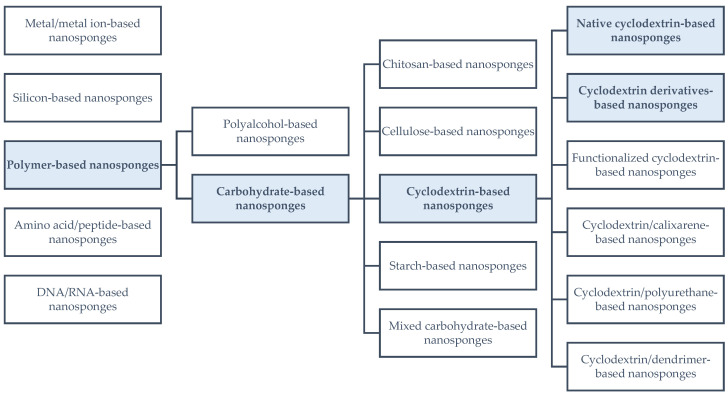
Family of nanosponge particles, with diverse representation of carbohydrate-based nanosponges. Marked boxes indicate the nanosponge groups included in this paper.

The last 50 years have witnessed the evolution of drug manufacturing, caused by implementation of numerous new formulating agents. As the 21st century arrived, a vigorously developing branch of nanotechnology arose, exerting a major influence on electronics, and also being used in biological applications. In the last 15 years, nanosponge particles (named for their sponge-like structure) have been intensively studied due to their possible applications, e.g., as drug carriers, toxin removal agents, or catalysts in organic syntheses [4,5,6,7,8]. Figure 1 presents the diversity of the nanosponge particle family [9,10,11,12,13,14,15,16]; however, most attention is directed towards cyclodextrin-based nanosponges, which were the first to be recognized from the entire group in the late 1990s [17,18].

Dextrin-based polymers evolved from the use of dextrins alone, which itself had growth and stagnation phases. The interest in the application of cyclic dextrins—cyclodextrins (CDs)—started with the works of Szejtli and coworkers in the 1980s and has continued up to the present day, with substantial interest in cyclodextrin-based polymers in the last fifteen years [19,20]. The purpose of this paper is to present the possibilities of using nanosponges as drug delivery systems, with the evaluation of their properties and possible relationships occurring between them, to better understand their pharmaceutical characteristics.

## 2. Cyclodextrin-Based Drug Delivery Systems—A Short Summary

### 2.1. Cyclodextrins

**Figure 2:**
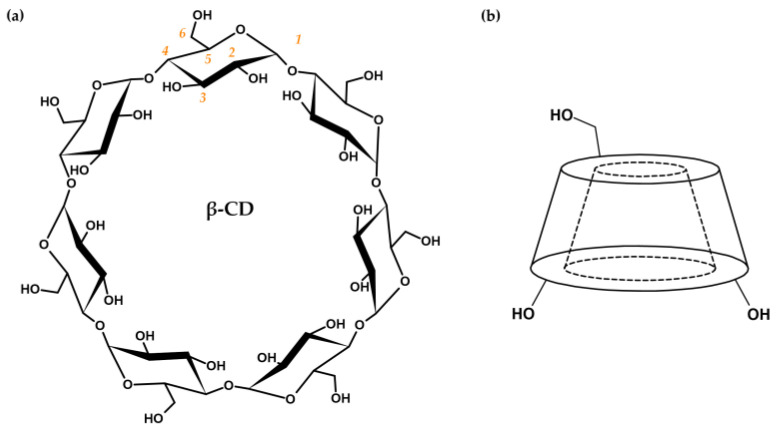
(**a**) Cyclodextrin structure based on β-CD with glucose units in chair conformation. The numbering order of carbon atoms of the glucose subunit is marked in orange. (**b**) Schematic representation of the CD shape, with labeled primary and secondary hydroxyl groups on the outer surfaces of the narrower and wider edges, respectively.

Cyclodextrins (hereinafter referred to as CDs) are cyclic oligosaccharides composed of α-d-glucopyranose units combined with α-(1→4)-glycosidic bonds. Native CDs are a product of the enzymatic degradation of starch, in which cyclodextrin glycosyltransferases produce cyclic oligomers consisting of six (α-CD), seven (β-CD, Figure 2a), or eight (γ-CD) glucose units [21,22,23,24]. Due to restricted rotation of glycosidic bonds and the chair conformation of each glucose unit, CDs possess the specific shape of a truncated cone with a hollow cavity (Figure 2b) [23,24,25,26,27].

The inner surface of CDs is lined with skeletal C-H groups and ethereal oxygen atoms that contribute to the lipophilic character of the cavity. The outer surface is composed of primary (at C_6_, narrower edge) and secondary (at C_2_ and C_3_, wider edge) hydroxyl groups that are responsible for the hydrophilic features of CDs (Figure 2b). Amphiphilic properties enable the binding of lipophilic drugs inside the CD cavities in the form of host–guest inclusion complexes [18,28,29,30]. Complexation of the drug and stabilization of the obtained complex are the result of conformational changes in the CD structure (steric relaxation of the ring) and the formation of various non-covalent bonds, i.e., van der Waals, hydrophobic, dipole–dipole, electrostatic interactions, hydrogen bonds, or dispersion forces [21,31,32,33,34]. On the other hand, the hydrophilic outer surface is responsible for increasing drug solubility through interactions with aqueous media via hydroxyl groups. Thus, CDs are great candidates for drug transport systems [21,23,24,25,26,30,31,35].

### 2.2. Cyclodextrin-Based Nanosponges

In addition to enhancing solubility and bioavailability, CDs have several drawbacks. Based on cavity size, CDs are usually capable of binding only one drug molecule (fully or partially), showing poor drug loading capacity. Additionally, relatively low stability constants of drug–CD complexes contribute to easy dissociation and release of their contents [18,21,26,36,37]. However, due to highly reactive hydroxyl groups, CDs can act as polyfunctional monomers [38,39]. Polymerization of CDs leads to the formation of cyclodextrin-based nanosponges (CDNSs) [17,18]. For CDNS synthesis, both natural and synthetic derivatives of cyclodextrins, e.g., 2-hydroxypropyl-β-CD (2-HP-β-CD), carboxymethyl-β-CD, sulfobutylether-β-CD, tosyl-β-CD, and a variety of methylated derivatives, can be used [25,29,30,40]. Nevertheless, β-CD is the most commonly used, owing to its non-toxic nature, low production costs, and highest stability constants in complexes with drugs [25,41,42,43]. Nanosponges are formed using linking agents called cross-linkers, which are highly reactive substances containing at least two active sites capable of covalent binding with hydroxyl groups of CDs. Cross-linking is a condensation polymerization reaction requiring the activation of CD hydroxyl groups by an electron-withdrawing group of the cross-linker. Activated hydroxyl groups are attacked with nucleophilic sites of the cross-linker, which usually binds with the primary hydroxyl group at C_6_ [4,39,44]. The topic of cross-linkers will be further explored later in this paper.

**Figure 3:**
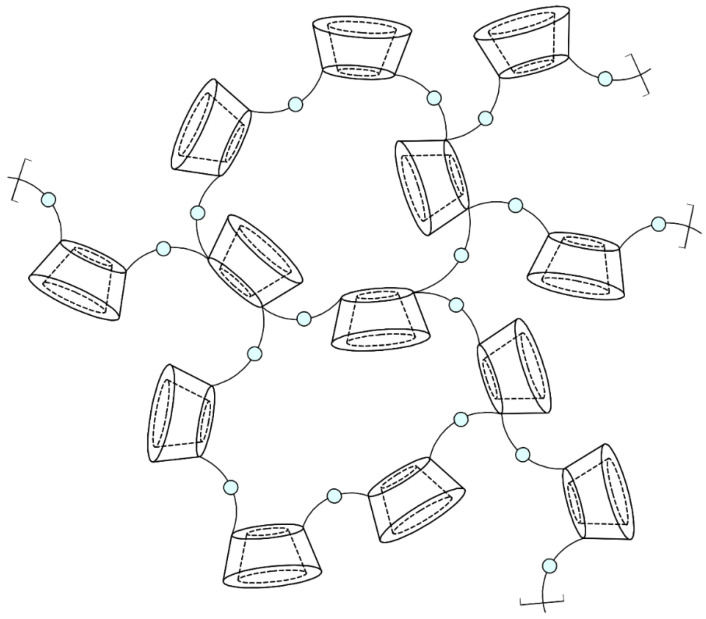
The structure of cyclodextrin-based nanosponges: Blue beads represent cross-linker molecules, which bind mostly to C_6_ hydroxyl groups on the narrower edge of the CD units (schematic structure, for visualization purposes only; degree of cross-linking not preserved).

Nanosponge particles are spherical and have a maximum diameter of 1 μm. The three-dimensional structure of CDNSs consists of parent CDs with their lipophilic cavities and nanochannels created between cross-linked CD units and cross-linkers (Figure 3). The hydrophilic properties of nanochannels are the result of the presence of multiple hydrophilic moieties of cross-linker molecules and free hydroxyl groups of CDs. Amphiphilic properties enable CDNSs to encapsulate a variety of drugs, both hydrophilic and lipophilic, in the form of non-inclusion or inclusion complexes. The former are made due to drug absorption on the CDNS surface, and they then migrate into hydrophilic channels. The latter are created through diffusion of the drug through the CDNS structure and inside the CD cavity [45,46,47,48]. The obtained drug–CDNS complexes present one of the highest stability constants for known non-covalent complexes (approx. 10^8^ M^−1^), much more stable in comparison with drug–CD complexes (50–2000 M^−1^) [49,50]. High stability could indicate the formation of irreversible complexes; however, in reality, the stability is disturbed in aqueous media, and the drug can be vigorously or gradually released from the CDNS. This phenomenon is the basis of the modifiable drug release properties of CDNSs, one of the most important properties of drug–CDNS complexes, which also reduces the likelihood of adverse effects [18]. Moreover, CDNSs are non-toxic, thermally stable up to 300 °C, chemically stable in environments of pH 1 to 11, and protect the drug against physical and chemical factors. Their production is simple, low-cost, and easy to scale up; they are compatible with most vehicles and excipients and are insoluble in most organic solvents and water, which is helpful in their purification and incorporation into CDNSs. As drug carriers, CDNSs can be formulated for oral, topical, parenteral, or inhalation use. Lastly, they are highly biodegradable—hydrolysis of CDNSs leads to parent CDs, which are completely degraded to glucose or maltodextrins by the colonic microflora. All of these properties make CDNSs versatile drug delivery systems with multiple possible medical applications [10,16,26,41,42,48,51,52,53,54].

However, further studies on using CDNSs as drug carriers should be performed, which, in turn, would earn the approval of the U.S. Food and Drug Administration (FDA) or the European Medicines Agency (EMA) for general use. At present, no drug–CDNS complex has received such approval. In 2023, there were 129 different formulations of drug–CD complexes recognized for medicinal general use, whereas ten years previously there were only 48 [55]. This looks promising in relation to CDNSs, which present better physicochemical properties required for good drug delivery systems as compared with CDs.

**Figure 4:**
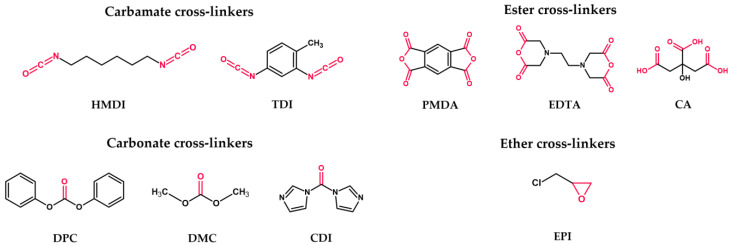
Chemical structures of the most frequently used cross-linkers. The donor moieties of each cross-linker group are labeled with red.

CD-based nanosponges can be divided into four generations [4,38]:

- *First generation*: plain nanosponges (Figure 4)—CDs polymerized with a cross-linker, used to obtain higher generations of CDNSs; cross-linker choice subdivides the generation into four types:
  a. *Carbamate nanosponges*—based on urethane compounds, e.g., hexamethylene diisocyanate (HMDI) or toluene-2,4-diisocyanate (TDI);
  b. *Carbonate nanosponges*—based on active carbonyl compounds, e.g., diphenyl carbonate (DPC), dimethyl carbonate (DMC), or 1,1′-carbonyldiimidazole (CDI);
  c. *Ester nanosponges*—based on di- or polycarboxylic acids or their dianhydrides, e.g., citric acid (CA), pyromellitic dianhydride (PMDA), or ethylenediaminetetraacetic dianhydride (EDTA);
  d. *Ether nanosponges*—based on epoxide-bearing cross-linkers, e.g., epichlorohydrin (EPI); since EPI was found to be toxic to humans, safety concerns were raised about its use in nanosponge production; however, it was proven that EPI-based CDNSs are non-toxic, owing to the fast hydrolysis of free EPI into harmless products during CDNS degradation [56].
- *Second generation*: modified nanosponges—equipped with chemical groups ensuring additional chemical or physical properties, such as fluorescence (ADME in vivo) or electric charge (binding polar drugs, increasing the stability of CDNS suspensions by inducing repulsive negative potential between particles).
- *Third generation*: stimulus-responsive nanosponges—sensitive to environmental stimuli such as temperature, pH, or redox potential, which can alter their physicochemical properties to trigger or enhance the release of the drug.
- *Fourth generation*: molecularly imprinted polymer nanosponges (MIP-NSs)—possessing binding sites with the imprinted structure of the drug, giving CDNSs high selectivity and affinity for target molecules.

Krabicova et al. [57] highlighted the possibility of the development of a fifth CDNS generation that, due to grafting the biological ligands on the CDNS surface, would bind with specific molecular targets to improve the drug’s bioavailability and the therapeutic effect. Apart from drug carrier applications, CDNS properties have been applied to other medicinal uses, such as protein [58] and enzyme [59] delivery systems, taste-masking agents [60], biosensors [61,62], gas transporters [63,64,65], and in fabric functionalization procedures [66,67].

### 2.3. Methods of CDNS Synthesis

There are four basic techniques of CDNS synthesis [9,29,31,68]:

- *The melting method*, during which CDs and cross-linkers are combined by melting at a temperature of up to 130 °C for several hours—longer synthesis results in nanosponges with a higher cross-linking degree;
- *The solvent method*, in which CDs and cross-linkers are solubilized in organic solvents, usually *N,N*-dimethylformamide (DMF) or dimethyl sulfoxide (DMSO), the use of which is justified by solvation of CDNSs and drug release from CD in an aqueous environment; cross-linking is initiated at a temperature ranging from 10 °C to the reflux temperature of the solvent and can last for up to 48 h;
- *The ultrasound-assisted method*, which uses ultrasound as a homogenizer in the presence of a solvent or in a solvent-free environment; the reaction proceeds at 90 °C for five hours in an ultrasonic bath filled with water;
- *The microwave-assisted method*, where microwave irradiation plays the role of homogenizing agent, reducing the reaction time and ensuring proper homogenization of substrates.

Each cross-linker type requires a different synthesis approach. The use of dianhydrides requires the addition of triethylamine (Et_3_N) as a catalyst. Et_3_N is a nucleophile and helps to reorganize the cross-linker structure to obtain free ester and carboxyl groups that can react with the hydroxyl groups of CDs [69,70,71]. Since CDI might react with only one CD, fresh CDI-based nanosponges still contain slight amounts of active imidazolyl carbonyl groups, resulting in the formation of dead ends in the polymer structure. This can be avoided by treating CDNSs with water, which enables an almost full elimination of imidazolyl carbonyl groups in 8 h [72]. A similar situation occurs for DPC and phenol moieties as residuals of the synthesis process.

Every method of synthesis includes a suitable purification process, where the various unreacted substrates, byproducts, solvents, and impurities are removed [38]. The most frequently used purification method is Soxhlet extraction using water or organic solvents (ethanol or acetone) [26]. Water removes unreacted CDs, while the ethanol/acetone extraction eliminates the unreacted cross-linker and impurities [29,72].

The four commonly used synthesis approaches have easily modifiable parameters, creating transparent differences between synthesis runs. More sophisticated methods are also in use and worth mentioning; however, they are often related to more complex chemistry or require the application of specialized equipment. The emulsion solvent diffusion method relies on the emulsification process, where the internal phase (consisting of a mixture of drug and CD in a volatile solvent) is added dropwise to the external phase (emulsifying solution) with continuous stirring until evaporation of the internal-phase solvent [9,73]. In the interfacial condensation method, the nanosponges are formed at the interface of a strongly alkaline phase (usually potassium hydroxide solution with pH > 10) with dissolved CD and an organic phase containing the cross-linking agent [74,75]. A more environmentally friendly approach is provided by mechanochemical synthesis, where no solvent is used. Instead, the synthesis is driven by the application of mechanical forces, which provide the energy necessary for the formation of chemical bonds. These types of syntheses are carried out by ball-milling (on a small scale) or using a twin-screw extruder (on a large scale) [72,76].

The synthesis of CDNSs requires the use of appropriate amounts of CD and the cross-linker. The precise mass of each substrate is not significant, but their molar ratio gives valid information about chemical composition. In this paper, the CD/cross-linker ratio will be depicted as 1:*n*, where *n* is the number of cross-linker moles per CD mole, generally being even, e.g., 1:2, 1:4, etc. Furthermore, shorthand notation will be used for different CDNSs, where the CD and cross-linker types will be followed by the CD/cross-linker ratio (if needed) in parentheses, e.g., β-CD:CDI (1:4).

The structure of synthesized CDNSs can vary at the molecular level, due to different 1:*n* values. The appropriate synthesis method and conditions are key factors to obtain more rigid structures, related to the higher degree of probe crystallinity. The creation of conditions appropriate for an efficient polymerization process results from even homogenization of the mixture. Thus, using methods with high-intensity mixing, e.g., ultrasound- or microwave-assisted methods, one could produce nanosponges with a highly organized and rigid crystalline structure, whereas basic methods like the melting method do not provide a suitable degree of homogenization, resulting in the formation of CDNSs with a geometrically less-organized, paracrystalline structure [77].

## 3. Revision of CDNSs’ Physicochemical Properties and Their Mutual Relationships

Knowing the basics of structure and macroscopic properties, we wanted to focus on the microscopic and pharmaceutically important characteristics of CDNSs. For this purpose, the results of studies on different drug–CDNS complexes were analyzed. We wanted to focus on simple CDNSs, so no complexes with functionalized, grafted, or co-polymer CDNSs were included in the present review (see Figure 1). Moreover, we wanted to focus on complexes of substances established as drugs, so we did not take into account any articles studying complexes with non-drug, mostly plant-based substances. Also, hydrogel formulations were not included, since they present different properties than standard CDNSs. The most important data about the selected complexes are presented in Table 1.

### 3.1. Drug Loading

The drug loading procedure is simple—the excess of a drug is added to an aqueous suspension of CDNS and stirred for the time needed for drug–CDNS complexes to form. Then, the unreacted free drug and CDNS are removed by washing or centrifugation procedures. To obtain a fine powder, freeze-drying or solvent evaporation methods are used, after which the drug–CDNS complexes are ready for further physicochemical evaluation [9,47]. The size of the CD cavities and CDNS channels enables the encapsulation of small molecules (up to 500 Da), but the number of available drug-binding moieties compensates for this disadvantage, making CDNSs drug carriers with high drug loading capacity [41,78]. Usually, drugs are incorporated into CDNSs at a precise mass ratio, named for the purpose of our work as *D:NS*, which is a new parameter describing nanosponges.

Drug loading can be described using two parameters—encapsulation efficiency and loading capacity.

Encapsulation efficiency (EE) describes the drug content loaded into the CDNS:

(1) $$EE=\frac{m_{loaded}}{m_{total}}\times 100\%$$

where m_loaded_ is the mass of the drug loaded into the CDNS and m_total_ is the mass of the drug used in the drug loading procedure.

Loading capacity (LC) describes the amount of encapsulated drug in the total mass of the drug–CDNS complex:

(2) $$LC=\frac{m_{loaded}}{m_{complex}}\times 100\%$$

where m_loaded_ is the mass of the drug loaded into the CDNS and m_complex_ is the mass of the obtained drug–CDNS complex.

**Table 1:** Characterization of selected drug–CDNS complexes.

Symbol	CDNS	Drug	Synthesis Method	1*:n*	*D:NS*	Particle Diameter (nm)	Zeta Potential (mV)	Drug Content (%)	Ref.
**1**	β-CD:CDI	Bortezomib	S	1:2	N/A	119.6	−21.6	51.5 ^a^; 20.6 ^b^	[79]
				1:4	N/A	241.3	−15.8	72.0 ^a^; 28.2 ^b^	
**2**	β-CD:CDI	Flutamide	S	1:2	N/A	82.53 ± 42.32	−43.0 ± 9.89	36.05 ^a^; 6.0 ^b^	[80]
				1:4	N/A	99.10 ± 22.15	−24.1 ± 10.1	56.5 ^a^; 9.4 ^b^	
**3**	β-CD:CDI	Piroxicam	S	1:2	1:2	322 ± 10.26	17 ± 2.34	35.22 ± 3.12 ^a^	[81]
					1:4	328 ± 16.05	20 ± 1.21	37.24 ± 1.25 ^a^	
					1:8	330 ± 14.04	15 ± 3.31	36.63 ± 5.27 ^a^	
				1:4	1:2	347 ± 15.12	15 ± 2.12	50.93 ± 3.02 ^a^	
					1:4	351 ± 13.02	12 ± 3.10	49.80 ± 6.01 ^a^	
					1:8	349 ± 17.16	10 ± 1.11	52.33 ± 5.22 ^a^	
				1:6	1:2	351 ± 20.16	13 ± 1.23	52.33 ± 5.22 ^a^	
					1:4	345 ± 18.20	15 ± 2.60	57.52 ± 4.53 ^a^	
					1:8	355 ± 15.12	14 ± 2.00	65.2 ± 4.14 ^a^	
				1:8	1:2	362 ± 14.06	17 ± 1.05	79.13 ± 4.33 ^a^	
					1:4	359 ± 18.10	10 ± 3.22	71.90 ± 1.22 ^a^	
					1:8	369 ± 14.01	15 ± 2.31	72.81 ± 3.12 ^a^	
**4**	β-CD:CDI	Sulfamethoxazole	S	1:2	N/A	116.3 ± 0.5	10.6 ± 0.6	31.4 ± 0.1 ^a^	[82]
				1:3	N/A	122.9 ± 8.8	12.4 ± 0.3	52.8 ± 2.8 ^a^	
				1:4	N/A	108.3 ± 15.1	12.7 ± 0.3	61.1 ± 0.2 ^a^	
**5**	β-CD:DPC	Baricitinib	M	1:1.5	N/A	261.9 ± 6.9	−15.8 ± 2.4	28.7 ± 2.3 ^a^	[83]
				1:3	N/A	306.1 ± 9.4	−21.2 ± 1.6	32.3 ± 4.8 ^a^	
				1:4.5	N/A	345.8 ± 4.7	−25.3 ± 3.2	79.1 ± 1.6 ^a^	
				1:6	N/A	864.3 ± 8.5	−19.2 ± 5.3	82.6 ± 8.3 ^a^	
**6**	β-CD:DPC	Febuxostat	S	1:4	N/A	224.76 ± 0.85	−21.5	88.42 ± 0.17 ^a^	[84]
				1:6	N/A	264.80 ± 0.54	−24.8	90.55 ± 0.43 ^a^	
				1:8	N/A	256.41 ± 0.89	−27.4	98.44 ± 0.21 ^a^	
				1:10	N/A	305.6 ± 0.78	−32.3	100 ± 0.05 ^a^	
**7**	β-CD:TDI	Naproxen	S	1:2	N/A	450.63 ± 10.07	−37.12 ± 3.01	80–95 ^a^ (for all 1:*n*)	[85]
				1:4	N/A	430.41 ± 9.10	−38.53 ± 3.22		
				1:8	N/A	378.45 ± 7.54	−39.45 ± 4.71		
**8**	2-HP-β-CD:DMC	Clotrimazole	M	1:2	1:1	N/A	N/A	15.34 ± 0.49 ^a^	[86]
					1:2	N/A	N/A	39.76 ± 0.83 ^a^	
				1:4	1:1	N/A	N/A	51.46 ± 0.91 ^a^	
					1:2	N/A	N/A	62.89 ± 1.19 ^a^	
				1:8	1:1	N/A	N/A	74.23 ± 0.74 ^a^	
					1:2	455.6 ± 11	−21.42 ± 1.3	85.12 ± 0.61 ^a^	
**9**	β-CD:DPC	Nifedipine	S	1:2	1:1	428 ± 1.06	−27	71.33 ± 1.62 ^a^	[87]
					1:2	422 ± 2.05	−30	72.26 ± 0.15 ^a^	
				1:4	1:4	430 ± 1.04	−25	78.4 ± 0.24 ^a^	
					1:6	422 ± 2.05	−35	77.26 ± 0.34 ^a^	
				1:6	1:8	444 ± 3.02	−32	62.11 ± 1.32 ^a^	
					1:10	446 ± 1.16	−30	60.23 ± 0.22 ^a^	
**10**	β-CD:DPC	Rilpivirine	S	1:2	N/A	391 ± 21.09	−24.67 ± 0.91	64.31 ^a^; 13.07 ^b^	[88]
				1:4	N/A	205 ± 17.39	−30.13 ± 2.71	91.54 ^a^; 18.63 ^b^	
				1:8	N/A	192 ± 11.23	−27.72 ± 1.49	85.77 ^a^; 16.43 ^b^	
**11**	β-CD:PMDA	Acetylsalicylic acid	S	1:2	N/A	59.53 ± 15.55	−35.45 ± 2.73	88 ^a^	[89]
				1:4	N/A	51.61 ± 13.47	−34.51 ± 0.95	91 ^a^	
				1:8	N/A	49.32 ± 11.23	−36.40 ± 1.80	73 ^a^	
**12**	β-CD:PMDA	Diclofenac sodium	S	1:1	1:1	N/A	N/A	65 ^a^; 33 ^b^	[90]
					1:2	407.2	−3.58	76% ^a^_;_ 38 ^b^	
				1:2	1:1	N/A	N/A	70% ^a^; 35 ^b^	
					1:2	333.1	−33.8	64% ^a^; 21 ^b^	
				1:4	1:1	N/A	N/A	41% ^a^; 19 ^b^	
					1:2	867.4	−17.5	30% ^a^; 13 ^b^	
**13**	β-CD:PMDA	Rosuvastatin calcium	S	1:4	1:1	612.00 ± 12.67 (avg. for both *D:NS*)	−56.80 ± 5.19 (avg. for both *D:NS*)	80.97 ± 0.40 ^a^; 40.48 ± 0.20 ^b^	[91]
					1:2			85.71 ± 0.85 ^a^; 28.55 ± 0.31 ^b^	
				1:6	1:1	275.50 ± 1.21 (avg. for both *D:NS*)	−61.90 ± 2.56 (avg. for both *D:NS*)	84.00 ± 0.50 ^a^; 42.00 ± 0.25 ^b^	
					1:2			88.87 ± 0.95 ^a^; 29.59 ± 0.31 ^b^	
				1:8	1:1	541.55 ± 16.80 (avg. for both *D:NS*)	−55.70 ± 4.73 (avg. for both *D:NS*)	79.85 ± 1.00 ^a^; 39.92 ± 0.50 ^b^	
					1:2			85.84 ± 0.50 ^a^; 28.61 ± 0.17 ^b^	
**14**	β-CD:CDI	l-DOPA	S	1:4	N/A	356.5 ± 5.3	−25.74 ± 0.36	90.5 ^a^	[92]
				1:8	N/A	381.7 ± 10.6	−26.21 ± 1.09	82.1 ^a^	
**15**	β-CD:DPC	5-Fluorouracil	M	1:4	N/A	N/A	−11.6 ± 3.59	64.05 ± 5.08 ^a^	[93]
				1:8	N/A	N/A	−24.3 ± 6.27	42.22 ± 4.71 ^a^	
**16**	β-CD:CDI	Paliperidone	S	1:4	N/A	307	−14.2	88.74 ^a^	[94]
				1:8	N/A	122	−8.74	89.44 ^a^	
**17**	2-HP-β-CD:DPC	Ciprofloxacin	U	1:0.5	N/A	66.7	N/A	99.99 ± 0.20 ^a^	[95]
				1:1	N/A	67.2	N/A	100 ± 0.30 ^a^	
				2:1	N/A	79.2	N/A	100 ± 0.07 ^a^	
**18**	β-CD:DPC	Camptothecin	M	1:2	1:4	907.3 ± 20.3 (avg. for all 1:*n*)	−20.71 ± 1.8 (avg. for all 1:*n*)	≈10% ^b^ (avg. for all 1:*n*)	[77]
				1:4	1:4				
				1:8	1:4				
			M + U	1:2	1:4	603.4 ± 20.2	−24.90 ± 2.1	21 ^b^	
				1:4	1:4	457.4 ± 15.7	−21.55 ± 1.7	37 ^b^	
				1:8	1:4	517.7 ± 12.4	−24.70 ± 2.4	13 ^b^	
**19**	β-CD:DPC	Clobetasol propionate	M	1:2	N/A	149.73 ± 33.45	−22.77 ± 0.38	6.21 ± 1.08 ^b^	[96]
				1:4	N/A	194.27 ± 49.24	−21.83 ± 0.95	12.34 ± 1.81 ^b^	
				1:6	N/A	210.77 ± 50.03	−21.83 ± 1.45	9.15 ± 1.36 ^b^	
				1:8	N/A	212.93 ± 41.92	−17.27 ± 2.70	6.8 ± 1.12 ^b^	
				1:10	N/A	138.97 ± 21.56	−20.27 ± 1.55	3.82 ± 0.70 ^b^	
**20**	β-CD:DPC	Irbesartan	M	1:4	1:1	200.7 (avg. for all *D:NS*)	−29 (avg. for all *D:NS*)	17 ^a^	[97]
					1:2			26 ^a^	
					1:4			33 ^a^	
**21**	β-CD:PMDA	Irbesartan	S	1:6	1:1	471.2 (avg. for all *D:NS*)	−34.9 (avg. for all *D:NS*)	25 ^a^	[97]
					1:2			38 ^a^	
					1:4			9 ^a^	
**22**	2-HP-β-CD:DPC	Ergotamine	S	N/A	1:2	1109.28 ± 11.7	15.9 ± 7.45	97 ^a^	[98]
					1:3	747.10 ± 8.3	13.7 ± 6.61	99 ^a^	
**23**	β-CD:CDI	Atorvastatin calcium	S	1:4	1:1	413.9 ± 17.3	−21.7 ± 0.90	17.9 ± 1.21 ^b^	[99]
					1:2	414.4 ± 15.4	−21.8 ± 1.09	28.3 ± 1.84 ^b^	
					1:3	408.7 ± 12.9	−22.1 ± 1.04	30.6 ± 1.28 ^b^	
					1:4	418.4 ± 15.2	−22.7 ± 0.85	34.1 ± 1.42 ^b^	
					1:5	423 ± 15.9	−22.6 ± 1.33	33.9 ± 1.16 ^b^	
					1:6	416.9 ± 12.5	−22.5 ± 1.02	33.7 ± 1.29 ^b^	
**24**	β-CD:DPC	Dexamethasone	S	1:4	N/A	N/A	N/A	N/A	[100]
**25**	β-CD:CDI	Acyclovir	S	1:4	N/A	403 ± 19	−25.0 ± 1.5	N/A	[101]
**26**	β-CD:SA	Acyclovir	S	N/A	N/A	415 ± 10	−28.2 ± 1.7	N/A	[101]
**27**	β-CD:DPC	Ibuprofen	S	1:2	N/A	521.2 ± 30	−17	47.59–88.13 ^a^ (for all synthesized complexes)	[102]
				1:4	N/A	453.2 ± 46	−25		
				1:6	N/A	611.7 ± 32	−27		
			U	1:2	N/A	501.1 ± 38	−21		
				1:4	N/A	431.2 ± 34	−27		
				1:6	N/A	583.4 ± 57	−29		
**28**	2-HP-β-CD:DPC	Ibuprofen	S	1:2	N/A	463.1 ± 51	−24		[102]
				1:4	N/A	398.2 ± 32	−27		
				1:6	N/A	520 ± 63	−29		
			U	1:2	N/A	400 ± 59	−25		
				1:4	N/A	296.8 ± 64	−30		
				1:6	N/A	484.2 ± 31	−32		
**29**	β-CD:DPC	Norfloxacin	M	1:2	N/A	N/A	N/A	80 ^c^	[103]
				1:4	N/A	N/A	N/A	76 ^c^	
				1:6	N/A	N/A	N/A	70 ^c^	
**30**	β-CD:CDI	Tamoxifen	S	1:2	N/A	400–600 (for all 1:*n*)	N/A	N/A	[104]
				1:4	N/A		N/A	N/A	
				1:8	N/A		N/A	N/A	
**31**	β-CD:DPC	Efavirenz	M	1:2	N/A	N/A	N/A	N/A	[105]
				1:4	N/A	N/A	N/A	N/A	
				1:8	N/A	N/A	N/A	N/A	
**32**	β-CD:CDI	Erlotinib	S	1:4	1:2	N/A	N/A	N/A	[106]
					1:4	372 ± 31	−32.07 ± 4.58	74.29 ± 6.81 ^a^	
					1:6	N/A	N/A	N/A	
**33**	β-CD:DPC	Griseofulvin	U	1:4	1:1	N/A	N/A	36.83 ± 0.7 ^b^	[60]
					2:1	N/A	N/A	20.20 ± 0.82 ^b^	
					3:1	N/A	N/A	21.00 ± 0.52 ^b^	
					4:1	N/A	N/A	22.90 ± 0.28 ^b^	
**34**	β-CD:CDI	Rilpivirine	S	1:4	N/A	457	−20.2	N/A	[107]
**35**	β-CD:PMDA	Rilpivirine	S	1:4	N/A	517	−21.55	N/A	[107]
**36**	β-CD:EDTA	5-Fluorouracil	S	1:8	N/A	N/A	−33.4 ± 4.32 (55.3%) −17.9 ± 3.29 (44.7%)	63.46 ± 3.48 ^a^	[93]
**37**	β-CD:DPC	Gabapentin	M	1:2	N/A	N/A	N/A	N/A	[108]
**38**	β-CD:PMDA	Meloxicam	S	1:8	N/A	350 ± 5.69	−29.3 ± 1.45	90.66 ± 4.82 ^a^	[109]
**39**	β-CD:DPC	Domperidone	S + U	1:6	N/A	195.68 ± 2.16	−9.17 ± 0.43	84 ± 4.2 ^a^; 42 ± 2.5 ^b^	[110]
**40**	β-CD:PMDA	Lansoprazole	S	1:4	1:1	204.9 ± 2.91	−30.32±2.37	89.23 ± 3.14 ^a^	[111]
**41**	β-CD:PMDA	Paclitaxel	N/A	1:4	N/A	316.8 ± 12.1	−28.4 ± 3.7	96.5 ^a^; 8 ^b^	[112]
**42**	β-CD:DPC	Dexamethasone	S + U	1:2	N/A	389.5 ± 21.4	−22.90 ± 1.1	3 ^c^	[113]
				1:4	N/A	688.6 ± 38.0	−26.55 ± 1.7	10 ^c^	
				1:8	N/A	657.9 ± 42.7	−24.70 ± 2.4	5 ^c^	
**43**	β-CD:CDI	Econazole nitrate	M	1:8	N/A	421 ± 6.82	N/A	71 ± 4.79 ^a^	[114]
**44**	β-CD:CDI	Dexamethasone	S	1:4	N/A	N/A	−30	35 ^a^	[115]
**45**	β-CD:CDI	Paclitaxel	S	1:4	N/A	450	N/A	N/A	[116]
**46**	β-CD:PMDA	Imiquimod	S	1:4	N/A	412.6 ± 10.2	−29.86 ± 1.45	96.5 ^a^; 14.2 ^b^	[117]
**47**	β-CD:DPC	Methotrexate	M	1:4	N/A	319 ± 6.7	−12.7 ± 4.7	36.63 ± 3.2 ^a^; 11.11 ± 3.2 ^b^	[118]
**48**	β-CD:DPC	Flurbiprofen	M	N/A	N/A	N/A	−30	15 ^c^	[45]
**49**	β-CD:DPC	Imatinib mesylate	M	1:1.5	N/A	N/A	−30.09 ± 0.81	94.61 ± 2.39 ^a^	[119]
**50**	β-CD:DPC	Paclitaxel	M	1:2	1:1	429 ± 45 (avg. for all 1:*n*)	N/A	N/A	[120]
				1:4	1:1		N/A	N/A	
				1:8	1:1		N/A	N/A	
**51**	β-CD:PMDA	Doxorubicin	N/A	1:4	N/A	310.4 ± 5.7	−29.8 ± 1.3	98.5 ^a^; 16.4 ^b^	[121]
**52**	β-CD:DPC	Doxorubicin	M	N/A	N/A	N/A	−30	4 ^c^	[45]

Abbreviations: 1:*n*, CD/cross-linker molar ratio; *D:NS*, drug/nanosponge molar ratio; M, melting method; S, solvent method; U, ultrasound-assisted; N/A, not available/applicable, ^a^ EE, ^b^ LC, ^c^ drug loading determined via HPLC.

To measure the EE and LC, the drug-loaded CDNS is dispersed in the appropriate solvent and sonicated to disrupt the drug–CDNS interactions. Then, the amount of the drug is determined by UV–vis spectroscopy or via HPLC methods [79,84,117].

Based on previous studies, the influence of different factors on drug loading properties can be established. Two major identified factors are *1:n* and *D:NS*, whose influence on EE and LC for different drug–CDNS complexes is shown in Table 2.

The relationship between EE and *1:n* shows four different natures:

- *Increase in EE with 1:n*—observed for β-CD:CDI complexes with bortezomib (**1**) [79], flutamide (**2**) [80], piroxicam (**3**) [81], and sulfamethoxazole (**4**) [82], β-CD:DPC complexes with baricitinib (**5**) [83] and febuxostat (**6**) [84], naproxen-loaded β-CD:TDI (**7**) [85], and clotrimazole-loaded 2-HP-β-CD:DMC (**8**) [86];
- *Maximum EE for specific 1:n*—observed for β-CD:DPC complexes with nifedipine (1:4) (**9**) [87] and rilpivirine (1:4) (**10**) [88], and for β-CD:PMDA complexes with acetylsalicylic acid (1:4) (**11**) [89], diclofenac sodium (1:2) (**12**) [90], and rosuvastatin calcium (1:6) (**13**) [91];
- *Decrease in EE with 1:n*—observed for l-DOPA-loaded β-CD:CDI (**14**) [92] and 5-fluorouracil-loaded β-CD:DPC (**15**) [93];
- *No influence of 1:n on EE*—observed for paliperidone-loaded β-CD:CDI (**16**) [94] and ciprofloxacin-loaded 2-HP-β-CD:DPC (**17**) [95].

For the LC–cross-linking degree relationship, two patterns occur:

- *Increase in LC with 1:n*—observed for β-CD:CDI complexes with bortezomib (**1**) [79] and flutamide (**2**) [80];
- *Maximum LC for specific 1:n*—observed for β-CD:DPC complexes with rilpivirine (1:4) (**10**) [88], camptothecin (1:4) (**18**) [77], and clobetasol propionate (1:4) (**19**) [96], as well as rosuvastatin calcium-loaded β-CD:PMDA (**13**) [91].

Three major cases can be noted for the EE–*D:NS* relationship:

- *Increase in EE with D:NS*—observed for irbesartan-loaded β-CD:DPC (**20**) [97], rosuvastatin calcium-loaded β-CD:PMDA (**13**) [91], and clotrimazole-loaded 2-HP-β-CD:DPC (**8**) [86];
- *Maximum EE for specific D:NS*—observed for irbesartan-loaded β-CD:PMDA (1:2) (**21**) [97];
- *No influence of D:NS on EE*—observed for ergotamine-loaded 2-HP-β-CD:DPC (**22**) [98].

The changes in LC with *D:NS* show two possible scenarios:

- *Maximum LC for specific D:NS*—observed for atorvastatin calcium-loaded β-CD:CDI (1:4) (**23**) [99];
- *Decrease in LC with D:NS*—observed for rosuvastatin calcium-loaded β-CD:PMDA (**13**) [91].

The influence of 1:*n* on drug loading parameters is diverse. Some patterns can be extracted from the existing research. A linear increase in EE and LC with 1*:n* is justified by the increase in the number of drug-binding sites owing to higher reticulation of the polymer network [79,80]. The maximum EE and LC at different 1*:n* ratios occur due to the satisfactory structure of CDNS for drug encapsulation, which can be disturbed by insufficient cross-linking and reduction in the formation of drug-binding sites (low 1*:n*) or excessive reticulation resulting in steric hindrances and polymer branching (high 1*:n*) [89,96,102]. A decrease in drug loading properties with 1*:n* can occur due to the formation of a highly dense network that, even at low 1*:n*, prevents the incorporation of the drugs at binding sites. In some cases, the relationship between loading properties and 1:*n* does not have a specific direction. Interestingly, a series of drug complexes with DPC-based nanosponges (**9**, **10**, **18**, and **19**) showed maximal EE at the same 1*:n* (1:4), which strongly implies that at this reticulation level this CDNS type binds the largest amount of drugs with respect to its mass [77,87,88,96]. On the other hand, complexes of PMDA-based nanosponges (**11**, **12**, and **13**) with three different drugs showed maximal loading properties at different 1*:n*, indicating that every drug requires a suitable cross-linking level for the most efficient encapsulation into PMDA-based CDNSs [89,90,91].

During drug loading, the *D:NS* needs to be optimal for the best encapsulation efficiency. When it comes to the increase in EE/LC with *D:NS*, the saturation concentration rises and more drug molecules are able to bind to the CDNS-binding sites [99]. Too high a concentration of the drug can lead to the formation of molecule aggregates, blocking the drug from binding with active sites of the CDNS. Also, in the binding site regions, steric hindrances occurring between drug molecules impede the encapsulation of the drug into the CDNS structure. Thus, *D:NS* needs to be high enough for the saturation of CDNS but low enough to avoid steric issues. Such a situation is achieved for CDNSs with maximal EE/LC for a specific *D:NS*. Rosuvastatin calcium-loaded β-CD:PMDA (**13**) [91] shows peculiar properties, since with the increase in the *D:NS* ratio the EE of rosuvastatin calcium increases while its LC decreases. This result indicates that reducing the drug concentration during drug loading enables encapsulation of a larger part of the used drug, but the overall contribution of the drug in the complex diminishes, which in the end is not the desirable outcome of manipulating the drug loading properties.

A series of other significant factors influencing drug loading were observed. A slight change in the crystallinity of the CDNS can lower the drug loading capacity and promote incidents of dose dumping, making crystallinity a very important factor during the CDNS formulation process. Differences in drug loading capabilities are a result of differences in the molecular structure of CDNSs. In crystalline CDNSs, the structure is rigid and has well-defined drug-binding sites. Destruction of the CDNS’s crystal structure leads to the loss of crystallographic sites for the drug, which is revealed by lower drug loading abilities, as observed for β-CD:DPC complexes with camptothecin (**18**) [77] and dexamethasone (**24**) [100].

The EE of acyclovir-loaded β-CD:CDI (**25**) and β-CD:succinyl anhydride (β-CD:SA) (**26**) nanosponges was equal to 38% and 69%, respectively, due to favorable electrostatic interactions between acyclovir and SA during drug loading [101]. Shoaib et al. [102] showed that ibuprofen-loaded β-CD:DPC (**27**) exhibited lower EE in comparison with ibuprofen-loaded 2-HP-β-CD:DPC (**28**), indicating the importance of the CD type chosen for synthesis. In addition, the same CDNS loaded with ibuprofen synthesized by the ultrasound method exhibited a higher EE value in comparison with a CDNS obtained by the solvent method. Drug loading also depends on the drug molecule—its chemical structure and physicochemical properties. Drug molecules that are too large cannot be fully encapsulated, leading to insufficient loading due to steric hindrances. Differences in EE could also be observed due to the various methods of drug inclusion in the CD cavity [98,109,122]. The drug structure could be pH-dependent, and its suitable matching for loading procedures could result in the formation of favorable interactions between the drug and the CDNS, as shown for norfloxacin-loaded β-CD:DPC (**29**) [103].

The abovementioned examples show that drug loading capability is sensitive to a variety of technical factors of synthesis and drug loading experiments, which is why proper preparation and execution of those processes allow one to obtain the desired CDNS with the optimal amount of the drug loaded. The conclusions presented here are based on single studies of different CDNSs, calling for further research into the relationships between those parameters.

### 3.2. Solubility Studies

Orally administered drugs are mostly absorbed in the small intestine, where they must pass the lipophilic membrane of the mucosa. Fick’s first law describes molecular transport through membranes via the following equation:

(3) $$J=P\times C_{aq}$$

where J is the drug flux through the membrane [mass/area/time], *P* is the permeability coefficient through the membrane and C_aq_ is the drug concentration on the membrane surface.

The permeability coefficient (*P*) can be expressed as follows:

(4) $$P=\frac{D\times K}{h}$$

where D is the diffusion coefficient of the drug within a membrane, K is the partition coefficient between the membrane and the aqueous exterior, and h is the membrane thickness.

The diffusion coefficient (D) is the product of the Stokes–Einstein equation:

(5) $$D=\frac{k_{B}T}{6\pi \eta r}=\frac{RT}{6\pi \eta rN}$$

where k_B_ is the Boltzmann constant, T is the temperature, η is the dynamic viscosity, r is the radius of the particle, R is the gas constant, and N is Avogadro’s number.

Equations (3)–(5) show that in order to be transported through the lipophilic membrane, the drug must have proper aqueous solubility (C_aq_), but also enough lipophilicity (K) and a relatively small particle diameter (r) [123]. Solubility also depends on the physical features of a given substance. Drugs in crystalline form are less soluble in aqueous media as compared with the amorphous form, due to strong attractive forces inside the crystal structure. Upon the loss of crystallinity, the strong forces are diminished, which results in increased solubility of the drug. The drug encapsulated into CDNS can lose its crystallinity, become fully amorphous, and form inclusion complexes with CD, or its crystallinity could be reduced, with partial transformation into the amorphous form and the formation of non-inclusion complexes inside the CDNS channels [124]. The studies on improving drug solubility by the formation of drug–CDNS complexes are the basis of research into CDNS complexes, which helps to understand the relationships between drug solubility and different CDNS properties.

Solubility studies evaluate how the inclusion of the drug into the CDNS changes its water solubility and dissolution kinetics. Usually, the amount of the solubilized drug is measured using UV–vis spectroscopy. This method makes use of the fact that the encapsulation of the drug chromophore inside the CDNS changes its UV–vis spectra due to a shift and broadening of the bands, which results in a shift in the maximum intensity of the spectra towards lower wavenumbers [105,118,125]. For a more direct approach, HPLC methods are also used [85]. Different experimental techniques lead to distinct solubility measurements. Solubilizing efficiency is obtained when the excess of the drug is added to the CDNS suspension during drug loading, whereas saturation solubility is acquired when the precise amount of the drug is added to create nanosponges with various (but known) values of *D:NS*. These are different expressions of solubility, since solubilizing efficiency describes a state where the drug concentration is significantly higher than that of the CDNS, which could create steric hindrances between drug molecules during loading, whereas saturation solubility makes it possible to evaluate the solubility of the drug in a saturated CDNS.

Similar to EE and LC, 1*:n* and *D:NS* can be identified as the most influential factors on the solubilizing properties of CDNSs (Table 3). The changes in 1*:n* may take two courses:

- *Increase in solubility with 1:n*—observed for β-CD:CDI complexes with piroxicam (**3**) [81], sulfamethoxazole (**4**) [82], and paliperidone (**16**) [94], naproxen-loaded β-CD:TDI (**7**) [85], and clotrimazole-loaded HP-β-CD:DMC (**8**) [86];
- *Maximum solubility for specific 1:n*—observed for β-CD:CDI complexes with atorvastatin calcium (1:4) (23) [99] and tamoxifen (1:4) (30) [104], complexes of β-CD:DPC with clobetasol propionate (1:4) (19) [96], irbesartan (1:4) (20) [97], ibuprofen (1:4) (27) [102], and efavirenz (1:4) (31) [105], irbesartan-loaded β-CD:PMDA (1:6) (21) [97], and ibuprofen-loaded 2-HP-β-CD:DPC (1:4) (28) [102].

The relationship between solubility and *D:NS* can result in the following outcomes:

- *Increase in solubility with D:NS*—observed for irbesartan-loaded β-CD:DPC (**20**) [97];
- *Maximum solubility for specific D:NS*—observed for erlotinib-loaded β-CD:CDI (1:4) (**32**) [106], β-CD:DPC complexes with rilpivirine (1:4) (**10**) [88], irbesartan (1:4) (**20**) [97], and griseofulvin (1:1) (**33**) [60], and irbesartan-loaded β-CD:PMDA (1:2) (**21**) [97].

Most of the aforementioned drug–CDNS complexes present maximum solubility at a given 1:*n*, implying that at a certain cross-linking level the saturation of drug binding occurs, preventing any further increase in drug solubility. This situation could be caused by the achievement of maximal loading capacity at a certain cross-linking level. This was presented by tamoxifen-loaded β-CD:CDI (**30**) [104], for which the solubility of the drug increased up to 1*:n* (1:4) and did not change after further reticulation to 1:*n* (1:8). This result could be explained by the fact that at lower cross-linking levels the access to the CD cavity is better and the drug is bound mostly in the form of inclusion complexes, while in denser networks, due to branching of the polymer, the cavities are less available, resulting in the formation of non-inclusion complexes to a degree that compensates the solubilizing properties coming from inclusion complexes. Interestingly, the maximum solubility increase for most of the mentioned drug–CDNS complexes occurs at 1:*n* 1:4, which might imply that this specific cross-linking level allows one to obtain the greatest solubilization efficiency, with particular emphasis on CDI- and DPC-based nanosponges.

Similarly to 1*:n*, the maximum solubility for most of the complexes occurred at the same *D:NS* value—1:4. This could be caused by saturation of the CDNS at a specific drug concentration during drug loading, which, when increased, does not provide further solubility enhancement due to steric hindrances at the binding site regions and the formation of drug aggregates. Analyzing the results concerning the influence of *D:NS*, one conclusion can be drawn—different CDNSs show the greatest solubilization efficiency for different values of *D:NS*, probably being the ratio at which the CDNS is fully saturated with the drug. In the case of griseofulvin-loaded β-CD:DPC (**33**) [60], the maximum solubility was obtained for *D:NS* (1:1) because the other complexes were synthesized with a large excess of the drug during drug loading. Thus, the solubility of griseofulvin could be further increased in complexes prepared with excess nanosponge, which is still awaiting confirmation.

Other types of factors influencing the solubility can also be identified. β-CD:CDI (**34**) was shown to be a worse solubilizer of rilpivirine as compared with β-CD:PMDA (**35**) [107]. Encapsulation of 5-fluorouracil into β-CD:DPC (**15**) ended with no change in drug solubility, whereas that into β-CD:EDTA (**36**) resulted in a slight increase in solubility [93]. This increase could be related to the formation of strong hydrophilic channels due to the presence of free carboxyl groups, which serve as good binding sites for hydrophilic 5-fluorouracil. Complexes of ibuprofen with 2-HP-β-CD:DPC (**28**) showed higher solubility in comparison with those with β-CD:DPC (**27**) [102]. Furthermore, the abovementioned CDNSs synthesized with the use of ultrasound helped to obtain a higher solubilizing efficiency of ibuprofen in comparison with nanosponges obtained using the classic solvent method. In turn, these results show the importance of the CD type and the selected method of synthesis with respect to the solubilizing properties of CDNSs.

### 3.3. Phase Solubility Studies

During drug loading, one or more drug molecules can form complexes with the CD moiety. Nevertheless, 1:1 complexes of the drug and CD are most common, alongside 1:2 and 2:1 drug–CD complexes [30,126,127]. To establish the stoichiometry of drug–CD complexes, phase solubility studies are used, which also enable the calculation of the equilibrium constant of the complex. In the same manner, drug–CDNS complexes can be formed with a known, even *D:NS* ratio, thus enabling the use of phase solubility studies to define the influence of CDNSs on drug solubility and the equilibrium of complex formation, with the equilibrium (stability) constant (K_C_) of a given complex as follows:

(6) drug+CDNS⇄drug−CDNS

(7) $$K_{c}=\frac{\left[drug-CDNS\right]}{\left[drug\right][CDNS]}$$

The K_C_ value indicates the degree of supramolecular interactions between the drug and the CDNS [105]. Usually, the K_C_ value is composed of several equilibrium constants of a few different solubilizing processes happening due to different mechanisms of drug encapsulation [122].

**Figure 5:**
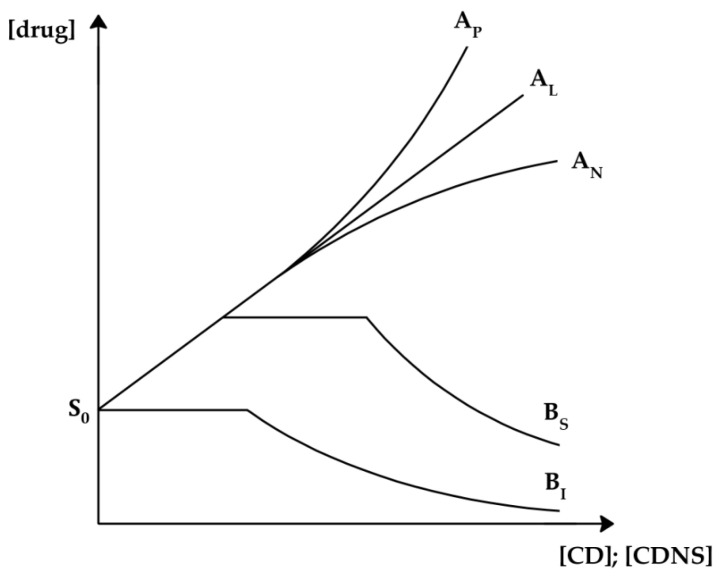
Schematic presentation of different phase solubility diagram types.

Phase solubility studies are a tool to evaluate the behavior of drug solubility with respect to the CDNS concentration [126]. The method developed by Higuchi and Connors provides the relationship between K_C_ and the intrinsic solubility of the drug (S_0_), i.e., the solubility of the drug in aqueous media with no solubilizer included [128,129]. A series of suspensions with known, increasing CDNS concentrations are treated with an excess of the drug, whose concentration is measured by UV–vis spectrometry using the calibration curve method. Figure 5 presents different possible types of phase solubility diagrams, which are divided into several types and subtypes [35,36,49,126].

The drug–CDNS complexes showing A-type curves possess partial water solubility, usually associated with water-soluble CD derivatives. A linear relationship between the drug and CDNS concentrations is represented by the A_L_-subtype curve, occurring for complexes with 1:1 stoichiometry. Linearity might be disturbed due to the formation of higher-order complexes. The positive deviation—A_P_-subtype curve—is characteristic of complexes of *D:NS* 1:2, 1:3, etc., where an increase in the amount of CDNS results in a better solubility improvement in comparison with 1:1 complexes, whereas the negative deviation—A_N_-subtype curve—occurs for *D:NS* 2:1, 3:1, etc., for which a further increase in the used nanosponge is hindered and approaches a plateau [35]. The B-type curve represents drug–CDNS complexes with reduced water solubility. The B_S_-subtype curve is typical of complexes with limited water solubility, for which the initial increase in solubility occurs, followed by precipitation of complexes, resulting in a curve plateau and a further decrease in solubility. Insoluble complexes present B_I_-subtype curves, the course of which is the same as that of B_S_-subtype curves, but without the initial solubility increase.

Predominantly, drugs and CDNSs form 1:1 complexes. The relationship between the stability constant for this complex type (K_1:1_) and S_0_ is described by the Higuchi–Connors relation [32]:

(8) $$K_{1:1}=\frac{Slope}{S_{0}(1-Slope)}$$

where Slope is the slope of the phase solubility diagram; S_0_, as the phase solubility diagram’s intercept, is considered to be a good estimate of solubility, but only when its value exceeds 1 mM [35]. For the linear drug-to-CD concentration relationship, Slope can be derived from Equation (9):

(9) $$Slope=\frac{S_{0}K_{1:1}}{S_{0}K_{1:1}+1}$$

Depending on the drug and CDNS types, a variety of phase solubility properties can be observed. The A_L_ type is the most common of them all, observed for β-CD:CDI complexes with sulfamethoxazole (**4**) [82] or rilpivirine (**34**) [107], complexes of β-CD:DPC with efavirenz (**31**) [105] and gabapentin (**37**) [108], and rilpivirine-loaded β-CD:PMDA (**35**) [107], which indicates the formation of inclusion complexes with established *D:NS* stoichiometry. However, deviations from linearity were also observed—for instance, in the case of diclofenac sodium-loaded β-CD:PMDA (**12**) [90], which is shown by the A_P_-type diagram, suggesting the formation of higher-order complexes.

Phase solubility properties can be influenced by the cross-linking level. Phase solubility studies of sulfamethoxazole-loaded β-CD:CDI (**4**) [82] showed increasing *K*_1:1_ values with 1*:n*, probably due to the formation of additional binding sites in the higher cross-linking environment. For griseofulvin-loaded β-CD:DPC (**33**) [60], an increase in 1:*n* leads to a change from linear phase solubility A_L_ (1*:n* 1:4) to negatively deviated A_N_ (1:*n* 1:6), implying that a further increase in 1:*n* does not provide better solubilization. On the contrary, it might lower the solubilizing efficiency of the CDNS, possibly due to polymer branching and hindrances occurring at the drug-binding sites. Phase solubility diagrams of ibuprofen complexes with β-CD:DPC (1:4) (**27**) and 2-HP-β-CD:DPC (1:4) (**28**) [102] appeared to be of the B_S_ and A_L_ subtypes, respectively. Furthermore, the *K*_1:1_ of the latter was higher in comparison with natural CD-based CDNSs, indicating that the solubility enhancement of the drug depends on the CD type used in the CDNS synthesis, which plays an important role in altering phase solubility and the inclusion/non-inclusion complex formation ratio.

During drug loading, drugs can form dimers, trimers, or oligomers that are unable to form inclusion complexes with CDs. Then, *S*_0_ does not make an equal intercept, which affects the value of *K*_1:1_. However, another parameter can be derived from the Higuchi–Connors relation (Equation (9)), called complexation efficiency (CE) [130,131,132]:

(10) $$CE=K_{1:1}S_{0}=\frac{\left[drug:CDNS\right]}{\left[CDNS\right]}$$

CE is a more accurate measurement of the cyclodextrin’s solubilizing effect, which is not dependent on either *S*_0_ or the intercept [32,131]. CE can change in the same manner as solubility, e.g., for sulfamethoxazole-loaded β-CD:CDI (**4**), CE increases with 1:*n* [82].

### 3.4. Morphology and Electrical Potential

The morphological evaluation of CDNS particles delivers information about their size, size distribution, shape, and porosity, which are important factors influencing the drug solubility, drug loading, proper formulation, and sustainability of the drug delivery system. The abovementioned parameters can be established using two methods—quantitative or qualitative approaches.

#### 3.4.1. Quantitative Approach

**Figure 6:**
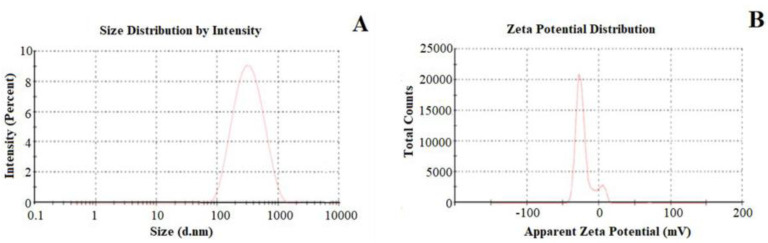
Graphical presentation of DLS measurements for the evaluation of (**A**) particle size and (**B**) zeta potential of baricitinib-loaded β-CD:DPC (1:4.5). Adapted from [83], licensed under CC BY 3.0.

The evaluation of particle size requires the use of dynamic light scattering (DLS). During the experiments, the laser beam is directed on the CDNS dispersion and scattered on the CDNS particles’ surface. The intensity of light measured by the detector changes constantly, since the particles are in constant Brownian motion. Smaller particles are characterized by faster motion and, thus, a higher-intensity disorder. The light intensity is measured in the time domain and can be converted into particle size by the autocorrelation function, which uses the Stokes–Einstein equation for diffusion of spherical particles (Equation (5)). Thus, DLS methods consider the temperature, viscosity, and refractive index (expressed by the diffusion coefficient) in particle size evaluation, where all of the particles are approximated to be spherical [31,54,78,133]. The results of DLS measurements are presented as intensity–particle size graphs (Figure 6A).

DLS experiments can be used to determine the polydispersity, expressed numerically as the polydispersity index (PDI), which presents the heterogeneity of the size of the particles in the probe. A high PDI value suggests strong dispersion of CDNS particle sizes, while a low PDI value shows small deviations from the average particle size and monodisperse nature of CDNSs [41,134]. Since the particles of helium are small enough to penetrate the cavities and channels of CDNSs, the helium displacement method and a helium pycnometer can be used to measure CDNSs’ porosity [119]. Interaction with any biological membrane strongly depends on the surface charge of the molecule, which is why zeta potential studies of drug–CDNS complexes are carried out to evaluate different physicochemical properties of CDNSs (most importantly, the stability of CDNSs in aqueous dispersions) [31,54,125]. The measurements are performed based on the Einstein–Smoluchowski relation, connecting the diffusion coefficient and electrical mobility of the particle:

(11) $$D=\frac{\mu_{q}k_{B}T}{q}$$

where D is the diffusion coefficient, μ_q_ is the electrical mobility of the charged particle, k_B_ is the Boltzmann constant, T is the temperature, and q is the electrical charge of the particle.

Similarly to particle size, the intensity–electrical mobility relationship can be established by DLS measurements and then transformed into an intensity–zeta potential graph (Figure 6B). Moreover, nanoparticle tracking analysis (NTA) can be used as another approach to obtain information about the particle size of CDNSs. This method uses an ultramicroscope to observe the Brownian motion of particles, the character of which enables the calculation of the hydrodynamic radius of the particles [135,136,137]. The NTA method is relatively new and still rarely used in classic nanosponge studies [138], but it offers a promising approach for future CDNS studies.

Lastly, the Brunauer–Emmett–Teller (BET) theory enables the evaluation of the surface area of CDNS particles. This method is implemented via evaluation of the pore volume of CDNS by adsorption of gas molecules, e.g., liquid nitrogen, which can then be used to calculate the pore diameter and, subsequently, the surface area [83,91].

#### 3.4.2. Qualitative Approach

The qualitative analysis of CDNS particles should be applied to expand the results of quantitative studies. Scanning electron microscopy (SEM) and transmission electron microscopy (TEM) are two imaging methods used to assess the shape, size, and morphology of CDNS particles [51]. High-resolution three-dimensional microscopic images help to understand how the changes in synthesis procedures and physicochemical properties influence the structure of CDNSs. SEM and TEM are used to obtain information about the porosity of CDNS [87]. Highly porous CDNSs have a regular and rigid structure with heterogeneous pore sizes, in comparison with less porous CDNSs [102]. Imaging methods are also used to confirm the polymerization reaction (formation of nanochannels) [105] or entrapment of the drug into the CDNS, which can be seen in images as the changes in CDNS particles’ pore structure [82,87,97,114] or the loss of drug crystallinity [73,81] caused by drug loading.

The abovementioned methods allow researchers to evaluate the microscopic parameters of CDNS morphology, which are often associated with the macroscopic properties of nanosponges. Two most important parameters are particle size and zeta potential.

#### 3.4.3. Particle Size Evaluation

Loading of the drug inside the CDNS structure usually results in a reduction in particle size below the size of the pure drug, like in the case of paliperidone-loaded β-CD:CDI (**16**) [94] and meloxicam-loaded β-CD:PMDA (**38**) [109], or else the particle size does not change significantly after encapsulation, as shown for domperidone-loaded β-CD:DPC (**39**) [110]. The decrease in particle size occurs owing to a better spatial arrangement of CDs due to cross-linking, which translates into a denser structure of the particles. This implies higher stability of complexes with CDNSs due to the greater surface area for drug encapsulation, which, on the other hand, leads to an increase in the wettability and solubility of the drugs [94,109]. Interestingly, particle size might increase after drug encapsulation, as shown by β-CD:PMDA complexes with lansoprazole (**40**) [111] and paclitaxel (**41**) [112]. In some cases, the particles could be too large to properly formulate the drug delivery system, due to a decrease in stability and insufficient drug loading. If needed, the particle size of CDNSs could be reduced using standard homogenization with a high rotation speed or high-pressure homogenization [107].

The particle size of CDNSs is strongly affected by 1*:n* (Table 4). Particle size can decrease with 1:*n*, as observed for naproxen-loaded β-CD:TDI (**7**) [85], rilpivirine-loaded β-CD:DPC (**10**) [88], acetylsalicylic acid-loaded β-CD:PMDA (**11**) [89], and paliperidone-loaded β-CD:CDI (**16**) [94], showing that higher reticulation enables dense packing of the polymer structure without branching. The minimum value of particle size for a given 1*:n* was observed for ibuprofen-loaded β-CD:DPC (1:4) (**27**) [102], β-CD:PMDA complexes with diclofenac sodium (1:2) (**12**) [90] and rosuvastatin calcium (1:6) (**13**) [91], and ibuprofen-loaded 2-HP-β-CD:DPC (1:4) (**28**) [102]. This phenomenon can be explained depending on the polymerization degree—for lower 1*:n*, the CDNS network is uncompleted, resulting in poor drug complexation and poor ability to form dense, organized structures, whereas for higher 1*:n*, polymer steric hindrances could lead to branching of the polymer structure. Particle size could also increase with the growth of 1*:n*, as shown by the principles of β-CD:CDI complexes with bortezomib (**1**) [79], piroxicam (**3**) [81], and l-DOPA (**14**) [92], and of β-CD:DPC complexes with baricitinib (**5**) [83] and nifedipine (**9**) [87], which are the result of destabilization of the polymer network, as mentioned previously.

A comparison of the particle size of drug–CDNS complexes obtained using the same CDNS type, but synthesized via different methods, could provide information about the direct influence of the method of synthesis on particle size. In all of the studied cases—camptothecin-loaded β-CD:DPC (**18**) [77] (melting/melting with ultrasound assistance); ibuprofen-loaded β-CD:DPC (**27**) and 2-HP-β-CD:DPC (**28**) [102] (melting/ultrasound assistance)—the particle size decreased due to the ultrasound used during the synthesis. Thus, ultrasound assistance could be required to lower the particle size, possibly due to fine homogenization of the substrates, which contributes to the formation of denser CDNS matrices.

### 3.5. Powder X-ray Diffraction (PXRD)

**Figure 7:**
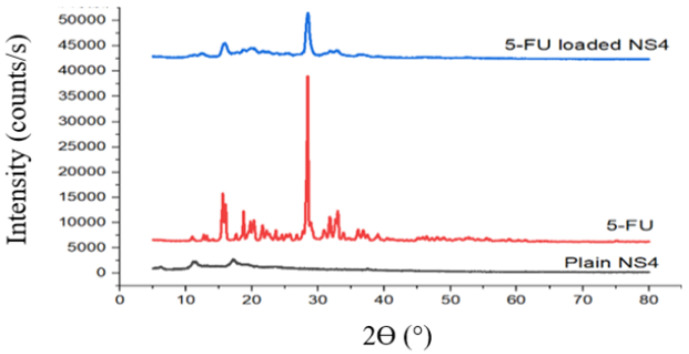
Comparison of PXRD patterns of (5-fluorouracil)-loaded β-CD:DPC (1:4), pure 5-fluorouracil, and pure β-CD:DPC (1:4). Adapted from [93], licensed under CC Attribution-NonCommercial-ShareAlike 4.0.

X-ray radiation directed at the sample is diffracted at the atoms of the sample. The diffracted waves are collected and converted into diffractograms, in which wave interferences can be seen as high and sharp peaks at specific 2*θ* degrees. Thus, the richer the diffractogram is in high-intensity signals, the more crystalline the examined probe. In this manner, PXRD experiments can be used to establish whether the obtained CDNSs are crystalline, paracrystalline, or amorphous. Usually, drugs are crystalline, while non-loaded CDNSs are partially or fully amorphous. This is caused by the presence of numerous free hydroxyl CD groups available for cross-linking, which leads to the formation of a highly disordered structure, unlike that of the crystals. The diffraction patterns of the drug, non-loaded CDNS, and loaded CDNS can be compared to describe the changes in the structural properties of the CDNSs after drug loading (Figure 7). Sharp peaks with high intensity versus half width at half-maximum (*I/hwhm*) are typical of crystalline solids, whereas low *I/hwhm* indicates poor crystallinity [100,101,108]. Since amorphous materials exhibit a higher Gibbs free energy and have a less rigid structure with more flexible lower energy bonds, non-crystalline CDNSs are capable of forming hydrogen bonds with water molecules to a greater extent as compared with crystalline nanosponges, resulting in improvements in the solubility of the drugs and drug loading [39,93,94,107,124]. 

Interaction of the drug with the CDNS disturbs its crystallinity, leading to partial or full conversion of the drug from a crystalline state to an amorphous state, which can be seen as partial or full reduction in the intensities of the drug signals. This could be observed for β-CD:CDI complexes with paliperidone (**16**) [94], tamoxifen (**30**) [104], erlotinib (**32**) [106], and rilpivirine (**34**) [107], β-CD:DPC complexes with 5-fluorouracil (**15**) [93], gabapentin (**37**) [108], domperidone (**39**) [110], and dexamethasone (**42**) [113], 5-fluorouracil-loaded β-CD:EDTA (**36**) [93], and β-CD:PMDA complexes with rilpivirine (**35**) [107] and meloxicam (**38**) [109]. Thus, PXRD is primarily used to confirm the formation of drug–CDNS inclusion complexes.

### 3.6. Thermoanalytical Methods

Thermal evaluation makes it possible to describe the behavior of CDNSs at higher temperatures, which lead to oxidation, polymorphic transformation, decomposition, melting, or evaporation of nanosponges [41]. Two main methods are used in such studies: differential scanning calorimetry (DSC), and thermogravimetric analysis (TGA).

#### 3.6.1. Differential Scanning Calorimetry

**Figure 8:**
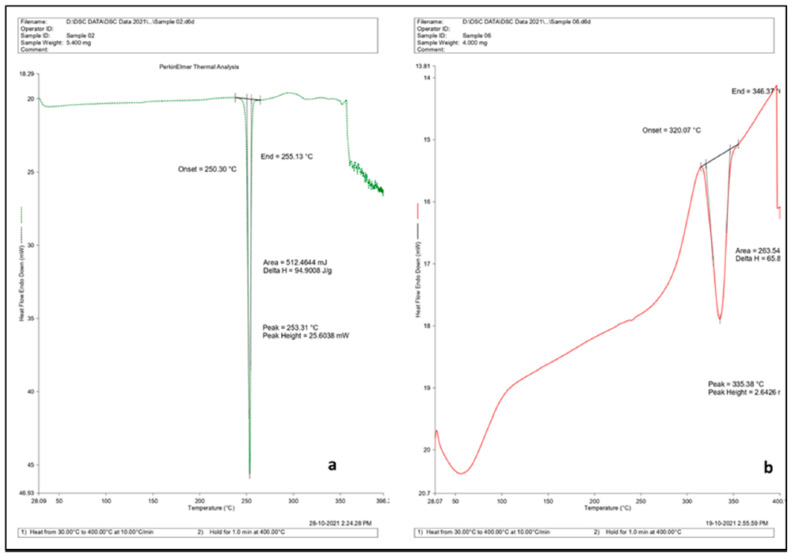
DSC thermograms of (**a**) pure domperidone and (**b**) domperidone-loaded β-CD:DPC. Disappearance of the endothermic signal of the drug indicates full incorporation of the drug into CDNS. A small signal at around 50 °C is most likely the result of water evaporation from CDNS. Adapted from [110], licensed under CC BY 4.0.

The DSC method consists in measuring the heat absorption of the probes, expressed as a difference in heat flux between the sample and a reference, or in the power supplied to them. It changes with a linear increase in temperature. The relatively poor thermal resistance of drugs is shown by endothermic peaks indicating their melting or decomposition at elevated temperatures. The formation of the drug–CDNS complexes is confirmed by comparison of thermograms of the drug, non-loaded CDNS, and loaded CDNS (Figure 8) [116,125]. Suppression of the drug’s endothermic peak shows a significant loss of crystallinity of the drug and its partial encapsulation into the CDNS. The disappearance of the endothermic signal of the drug means the complete loss of crystallinity due to full encapsulation and molecular dispersion inside the CDNS structure [91]. Table 5 presents the properties of encapsulation and thermal resistance of drug–CDNS complexes.

Not only the changes in melting point, but also its initial value, carry valuable information about CDNSs’ properties. Drugs with a high melting point form less stable complexes with CDNSs due to the rigidity of their structure and their poor ability to change shape, which is necessary during drug encapsulation [90]. A decrease in the melting point of the drug indicates a loss of crystallinity of the drug and a switch to an amorphous form after the formation of the complex with the CDNS [60,109,114,115]. The DSC thermograms make it possible to differentiate between the crystalline and paracrystalline probes, as in the case of camptothecin-loaded β-CD:DPC (**18**) [77], which showed no endothermic peak of the drug for the crystalline form, whereas for a paracrystalline CDNS the melting peak was present, suggesting that the drug remained in crystal form due to weaker interactions between the paracrystalline CDNS and the drug. Also, the DSC method can be used to observe the expulsion of water molecules from the β-CD cavity of insufficiently dried CDNSs. This can be seen as an endothermic peak around 100 °C, which is useful for verifying the anhydrous character of synthesized CDNSs [96,99,103].

#### 3.6.2. Thermogravimetric Analysis

The TGA method is used to analyze the thermal stability of CDNSs by means of weight loss with increasing temperature. Similarly to DSC, a comparison of thermograms of the pure drug, non-loaded CDNS, and loaded CDNS helps to evaluate the influence on thermal properties introduced by the formation of the CDNS–drug complex. If a significant weight loss of the loaded CDNS appears at higher temperatures than for the pure drug, the CDNS performs a protective function for the encapsulated drug, increasing the thermal resistance of the encapsulated drug [31,102,124]. The TGA method was used to assess the influence of CD type on thermal stability of CDNSs [70], and it also showed the possibility to verify the anhydrous character of CDNSs [96].

### 3.7. Vibrational Spectroscopy

The vibrational properties of molecules allow the use of spectroscopic methods for a fairly accurate description of their structure and its evolution. Thus, vibrational spectroscopy can be used to confirm cross-linking and CDNS formation [139]. The most noticeable change in Fourier-transform infrared spectroscopy (FT-IR) spectra confirming the successful synthesis of CDNSs is the appearance of signals assigned to characteristic chemical groups of cross-linkers. For instance, carbonate CDNSs show a stretching signal of carbonyl groups in the range of 1740–1770 cm^−1^ [82,88,108,110,114,118]; ester CDNSs show the stretching of ester carbonyl groups appearing between 1700 and 1720 cm^−1^, which are created during the opening of cyclic dianhydrides [62,90,91], whereas carbamate CDNSs show signals of amide-I-like carbonyl stretching (1630 and 1700 cm^−1^) and amide-II-like N-H bending (1550 cm^−1^) [29]. It was also found that, with the increase in 1:*n*, the intensity of characteristic CDNS signals rises, showing the possibility of controlling the cross-linking degree via FT-IR [90,140].

**Figure 9:**
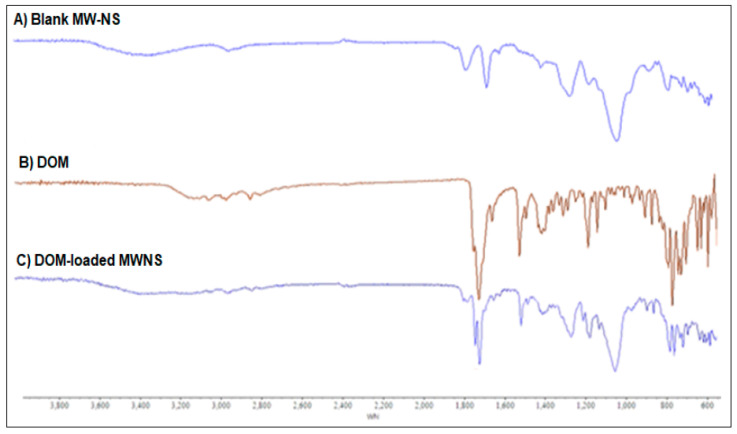
FT-IR spectra of (**A**) non-loaded β-CD:DPC, (**B**) pure domperidone, and (**C**) domperidone-loaded β-CD:DPC. The diminishing of drug signals, especially in the fingerprint region, can be observed after incorporation into nanosponges. Adapted from [110], licensed under CC BY 4.0.

FT-IR can be used to confirm the drug–CDNS complex formation. Examination of the spectra of the drug, CD, and the non-loaded and loaded CDNS enables the confirmation of the drug–CDNS complex formation. The changes occurring due to interactions between the drug and CDNS can be seen as diminishing and broadening of the characteristic signals of the drug [79,84,85,89,93,100,103,106,110,113,141,142]. Incorporation of the drug into the CDNS can also be noted in the fingerprint region (600–1400 cm^−1^), containing characteristic patterns for a given drug (Figure 9) [95,102,104,120,124]. Additionally, the appearance of a broad hydrogen bond signal in the frequency range of 3200–3500 cm^−1^ in the drug–CDNS complex spectra confirms the interaction of drug moieties and hydroxyl groups of CDs via H bonds, as was shown for β-CD:CDI complexes with flutamide (**2**) [80], atorvastatin calcium (**23**) [99], and tamoxifen (**30**) [104], clobetasol propionate-loaded β-CD:DPC (**19**) [96], and meloxicam-loaded β-CD:PMDA (**38**) [109].

Raman spectroscopy can be used to confirm the formation of drug–CDNS complexes, but this method is limited due to its lower precision and the lack of detection of significant signals that appear in the FT-IR spectra. However, these two methods complement one another, since the same signals might appear in Raman or FT-IR spectra with different intensity [100,143,144].

FT-IR and Raman signals comprise several sub-signals, each representing a different type of the given bond, which is characterized by different energy and a different signal maximum. The evolution of the positions and intensities of sub-bands carries the information about changes in the CDNS structure [145,146]. To extract the detailed quantitative information about the contribution of different bond types from the collective signal, band deconvolution can be performed using curve-fitting procedures. These methods use the second derivative test evaluation of the spectra as a guidance for finding the maxima of the individual sub-bands [147]. Based on experimental data, the curve fitting for each sub-band is carried out using the Voigt (FT-IR) or Gaussian/Lorentzian (Raman) functions, with the maximum acquired earlier. The intensities of the given sub-bands correspond to the population of the associated bond in the CDNS structure, which can be used in the evaluation of structure evolution occurring during drug loading. Usually, FT-IR data are used in deconvolution procedures of hydroxyl stretching (3000–3500 cm^−1^), whereas Raman spectra are used in those of carbonyl stretching (1700–1740 cm^−1^) [146,148]. The positions of the same bands in FT-IR and Raman spectra can differ due to being derived from different mathematical functions; thus, combined use of both spectroscopic methods is the most popular approach [149].

The deconvolution studies of the carbonyl stretching signal reveal two sub-bands—ester carbonyl stretching (ω_CO1_) at lower frequencies, and carboxyl carbonyl stretching (ω_CO2_) at higher frequencies [150]. The ratio of the intensities of the sub-bands (*I*_CO1_/*I*_CO2_) can be used as the descriptor of cross-linking/branching of the polymer; thus, band deconvolution of the carbonyl stretching signal enables a thorough examination of the structural evolution with the change in 1*:n*. For instance, for β-CD:EDTA [150] and β-CD:PMDA [146], *I*_CO1_/*I*_CO2_ increased with 1*:n* up to a maximum at 1*:n* 1:6, after which both ratios decreased. This result corresponds to the increase in the number of ester moieties with a cross-linking degree up to 1*:n* (1:6) (increase in *I*_CO1_, decrease in *I*_CO2_), after which further addition of the cross-linker leads to branching of the polymer (increase in *I*_CO2_, decrease in *I*_CO1_). For β-CD:CDI, the saturation point of cross-linking did not occur at 1*:n* (1:6), and the *I*_CO1_/*I*_CO2_ ratio increased up to 1*:n* (1:8) [149]. These results show that the critical 1*:n* value for which the polymerization reaction leads to the maximized cross-linking degree can differ in different nanosponge types.

In the case of the hydroxyl stretching bond, deconvolution leads to five sub-bands corresponding to four types of interactions, assigned to interstitial water molecules (ω_1_, ω_3_), intracavity water molecules (ω_5_), primary CD hydroxyl groups (ω_4_), and secondary CD hydroxyl groups (ω_2_) [143,146]. A shift in the maxima of sub-bands towards lower frequencies is related to the establishment of a stronger H-bond network, whereas a shift towards higher frequencies corresponds to a weakening of the H-bond structure, changes in the mobility of the sub-bands, and a decrease in their cooperativity. For instance, for β-CD:CDI, an increase in 1:*n* leads to a shift in all sub-bands towards higher wavenumbers, showing the destructive effect of cross-linking on the H-bond network [143]. With the increase in 1*:n*, the population of primary hydroxyl groups decreases (decrease in *I*_4_) with the increase in the population of secondary hydroxyl groups (increase in *I*_2_). These changes reflect the cross-linking reaction, since primary hydroxyl groups are mostly involved in the polymerization reaction, which results in a decrease in *I*_4_ [146,149]. The increase in *I*_2_ and the decrease in *I*_4_ with 1:*n* for β-CD:CDI is slower in comparison with β-CD:PMDA, which shows the differences in the stiffness and rigidity of the structure of different nanosponge types [146]. Overall, deconvolution studies of hydroxyl stretching sub-bands provide valuable information about the dynamics of the polymer.

### 3.8. Nuclear Magnetic Resonance (NMR)

Owing to their non-destructive and sensitive nature, NMR experiments are often used in the analysis of the structural features of different homogeneous solids [125]. The changes in chemical shift (*δ*) values or signal intensity in ^1^H NMR spectra indicate proton exchange between interacting moieties. Hence, NMR studies can confirm the formation of CDNSs [75], as well as drug–CDNS complexes, by comparison of the spectra obtained for drugs, CDs, and drug–CDNS complexes [31]. Such comparisons allow one to confirm the drug encapsulation, as shown for β-CD:DPC loaded with clobetasol propionate (**19**) [96], irbesartan (**20**) [97], and methotrexate (**47**) [118] and irbesartan-loaded β-CD:PMDA (**21**) [97]. NMR can also exclude the possibility of drug–CDNS complex formation. The ^1^H NMR experiments showed no significant signal shifts in the spectra of 5-fluorouracil-loaded β-CD:DPC (1:8) (**15**) and β-CD:EDTA (1:8) (**36**). However, complexation was confirmed for β-CD:DPC (1:4) (**15**), suggesting that 5-fluorouracil cannot be incorporated into CDs in highly cross-linked CDNSs [93]. Moreover, ^1^H NMR is also used to confirm the purity of synthesized CDNSs. The ^1^H NMR spectra confirmed the lack of impurities such as phenol, imidazole, and the unreacted cross-linker in β-CD:DPC in formulations loaded with rilpivirine (**10**) [88] and clobetasol propionate (**19**) [96], thus suggesting the correct course of polymerization and purity of the obtained formulations.

Since ^13^C NMR spectra show poor resolution and sensitivity, to obtain more precise information about the CDNS structure, some more sophisticated methods are incorporated. Magic-angle-spinning NMR (MAS NMR) enables the registration of the NMR spectra of solids with high resolution, due to averaging the anisotropy caused by the restricted movement of molecules [151]. For a further resolution increase, cross-polarization (CP) methods are applied that use the phenomenon of magnetization transfer, which enhances the intensity of low-abundance nuclei in the NMR spectra [152]. The ^1^H-^13^C magnetization transfer is used to obtain ^13^C CP/MAS NMR spectra carrying information about the CDNS structure [152]. For instance, a comparison of the ^13^C CP/MAS NMR spectra of β-CD and β-CD:CDI (1:3) shows no significant change in *δ* except for C_6_, for which two separate signals occur, implying greater participation of primary hydroxyl groups in the cross-linking process, as well as the existence of two cross-linking species that are cross-linked via two primary hydroxyl groups or via one primary group and one secondary hydroxyl group of CDs [143]. The registration of ^13^C CP/MAS NMR spectra at different contact times (CTs), needed for CP to occur, has led to the development of variable contact time (VCT) methods. CP data are fitted to the appropriate theoretical model corresponding to characteristic dynamic properties, resulting in the calculation of proton relaxation times (*T*_1*p*_) associated with different carbon atoms. The ^1^H-^13^C VCT CP/MAS NMR experiments carried out on the abovementioned β-CD:CDI (1:3) showed that, due to cross-linking, the mobility of hydrogen atoms of CDs increased in comparison with native crystalline β-CD, with one exception—hydrogen atoms of C_6_, which showed prolonged *T*_1*p*_, suggesting restrictions of movement due to cross-linking occurring on the primary hydroxyl groups of CDs [143]. Thus, NMR methods can be used to study the structure and dynamics of CDNSs.

### 3.9. Drug Release Studies

As previously mentioned, drug–CDNS complexes are characterized by high stability, which is impaired after exposure to environmental factors, leading to drug release. Release experiments are performed in multicompartment cells—the drug is placed in a donor compartment separated by a dialysis membrane from a receptor compartment, both containing solutions of various pH to simulate the behavior of CDNSs in different physiological environments [31,45]. During in vitro release, the aliquots are withdrawn at fixed times and replaced with a fresh portion of buffer solution. The amount of the drug in the aliquots is determined using spectroscopic (UV–vis), chromatographic (HPLC), or mixed chromatographic–mass spectrometry methods (LC-MS) [41]. The changes in drug concentration with time are shown in the drug release profiles, which usually present the cumulative drug amount released at a given time.

To illustrate the influence of multiple factors on the drug release process, insight into the results of drug release studies is necessary. Table 6 presents the simplified results of drug release studies of different drug–CDNS complexes.

The key factor responsible for the different release rates of the complexes is the type of drug-binding interaction. The drug molecules that are released from CDNSs the earliest are the superficial ones, arising as a result of the inability to form inclusion or non-inclusion complexes caused by the polymer network being too dense, resulting in hindered access to CD and nanochannels, or in low-reticulated networks that are incapable of binding the drugs in the form of non-inclusion complexes due to a very low amount of drug-binding sites. [102]. After that, the nanosponges release the drugs from the non-inclusion complexes, where they are bound within the nanochannels. Then, the drug is released from inclusion complexes, which are responsible for a prolonged drug release due to strong interactions with CD cavities and the necessity to diffuse through the CDNS network.

Differently bound drug molecules affect drug release profiles in different ways. Weakly bound drugs can contribute to the fast release of the drug in the early stages of drug release, known as initial burst release, after which further release is continued in a prolonged manner. This phenomenon was observed for β-CD:CDI complexes with piroxicam (**3**) [81] and atorvastatin (**23**) [99], along with ibuprofen-loaded 2-HP-β-CD:DPC (**28**) [102], due to the release of drug molecules adsorbed at the surface of the CDNS. Simultaneously, for the β-CD:DPC complexes with febuxostat (**6**) [84] and nifedipine (**9**) [87], the initial burst was related to the presence of non-inclusion complexes. The occurrence of the initial burst could be influenced by 1:*n*, since it was observed for bortezomib-loaded β-CD:CDI (1:2), but not for 1:*n* (1:4) (**1**) [79], suggesting better encapsulation of the drug in the form of inclusion complexes at a higher cross-linking level. However, more studies are necessary to fully understand the course and genesis of the initial burst process. Neglecting the different relationships between the drug release and CDNS properties, both fast and slow release have their advantages. Accelerated release helps to achieve a therapeutic concentration of the drug in comparison with slow release, whereas a prolonged release could decrease the risk of adverse effects that often occur during a rapid release [77,85,89,98,109,118].

The incorporation of drugs into CDNSs alone can lead to changes in the cumulative drug release observed at the same time, which translates into accelerating or slowing down the drug release rate (Table 7). An increase in cumulative drug release as compared with the pure drug was observed for β-CD:CDI complexes with bortezomib (**1**) [79], flutamide (**2**) [80], atorvastatin calcium (**23**) [99], and rilpivirine (**34**) [107], β-CD:DPC complexes with baricitinib (**5**) [83], rilpivirine (**10**) [88], irbesartan (**20**) [97], dexamethasone (**24** and **42**) [100,113], efavirenz (**31**) [105], erlotinib (**32**) [106], and domperidone (**39**) [110], β-CD:PMDA complexes with rosuvastatin calcium (**13**) [91], irbesartan (**21**) [97], rilpivirine (**35**) [107], and imiquimod (**46**) [117], and 2-HP-β-CD:DPC complexes with ibuprofen (**28**) [102], due to increased wettability and improvement of the water solubility of the drug [80,88,97,99,107], reductions in drug crystallinity [88,97] and particle size [88,97,100,117], and increases in the surface area [83,88] resulting from encapsulation into CDNSs. On the other hand, a decrease in the amount of drug released was observed for β-CD:CDI complexes with paliperidone (**16**) [94], econazole nitrate (**43**) [114], and dexamethasone (**44**) [115], β-CD:DPC complexes with febuxostat (**6**) [84], clobetasol propionate (**19**) [96], methotrexate (**47**) [118], flurbiprofen (**48**) [45], and imatinib mesylate (**49**) [119], β-CD:TDI complexes with naproxen (**7**) [85], and 2-HP-β-CD:DPC complexes with ergotamine (**22**) [98], which was primarily caused by the formation of inclusion complexes. Rarely, incorporation of drugs into CDNSs does not influence their release profile. This particular situation occurred after the incorporation of 5-fluorouracil into β-CD:DPC (**15**) and β-CD:EDTA (**36**), possibly due to the low stability and short lifetime of the complex with β-CD, resulting from the inability to form stable interactions between the drug and CDNS [93]. At this point, it should be remembered that not only reactivity, but also the size, electric charge, and lipophilicity of the drug molecule matter during the formation of complexes; thus, they are also valid factors in drug release.

**Table 6:** Results of drug release studies and kinetic profile evaluation of drug–CDNS complexes.

Symbol	CDNS	Drug	1:*n*	Cumulative Drug Release/(Time)				Kinetic Model of Drug Release		Diffusion Properties	Ref.
				Drug-CDNS		Initial Burst Release	Pure Drug				
**1**	β-CD:CDI	Bortezomib	1:2	78% (72 h)		(4 h) (for all 1*:n*)	25% (72 h)	N/A		N/A	[79]
			1:4	58% (72 h)							
**2**	β-CD:CDI	Flutamide	1:2	≈55% (1 h)	100% (3 h)	Not observed	≈20% (1 h) ≈25% (3 h)	N/A		N/A	[80]
			1:4	≈65% (1 h)							
**3**	β-CD:CDI	Piroxicam	1:2	N/A		20.23–30.45% (30 min) (for all formulations)	5.23 ± 3.0% (30 min)	H (all *D:NS*)		Fickian diffusion (for all formulations)	[81]
			1:4	45.33 ± 2.6% (2 h, *D:NS* 1:2) 48.41 ± 1.44% (2 h, *D:NS* 1:4) 52.50 ± 1.5% (2 h, *D:NS* 1:8)				H (*D:NS* 1:2) H (*D:NS* 1:4) H/KP (*D:NS* 1:8)			
			1:6	48.23 ± 3.0% (2 h, *D:NS* 1:2) 55.67 ± 2.66% (2 h, *D:NS* 1:8)				KP (*D:NS* 1:2) H/KP (*D:NS* 1:8)			
			1:8	86.15 ± 3.0% (2 h, *D:NS* 1:2) 95.1 ± 2.39%, (2 h, *D:NS* 1:4) 89.64 ± 2.2% (2 h, *D:NS* 1:8)				KP (*D:NS* 1:2) H (*D:NS* 1:4) H (*D:NS* 1:8)			
**4**	β-CD:CDI	Sulfamethoxazole	1:2	100% (4 h)		Not observed	N/A	H (for all 1*:n*)		Fickian diffusion (for all 1*:n*)	[82]
			1:3	64% (24 h)							
			1:4	54% (24 h)							
**5**	β-CD:DPC	Baricitinib	1:4.5	61 ± 4.2% (3 h)		Not observed	40 ± 6.4% (3 h) 100% (under 12 h)	KP		Anomalous transport	[83]
**6**	β-CD:DPC	Febuxostat	1:4	78.05–93.36% (8 h) 100% (24 h) (for all 1:*n*)		30.33 ± 0.96–53.07 ± 0.87% (2 h) (for all 1:*n*)	100% (3 h)	H		Fickian diffusion (for all 1*:n*)	[84]
			1:6					H			
			1:8					H			
			1:10					KP			
**9**	β-CD:DPC	Nifedipine	1:4	N/A		80% (4 h)	N/A	KP (*D:NS* 1:4)		Anomalous transport	[87]
**10**	β-CD:DPC	Rilpivirine	1:4	≈ 60% (40 min) ≈ 85% (1 h)		Not observed	≈30% (40 min) ≈40% (1 h)	H		Anomalous transport	[88]
**11**	β-CD:PMDA	Acetylsalicylic acid	1:2	83–91% (24 h) (for all 1:*n*)		Not observed	N/A	N/A		N/A	[89]
			1:4								
			1:8								
**12**	β-CD:PMDA	Diclofenac sodium	1:1	≈ 80% (1 h) (for all 1*:n*)		Not observed	N/A	N/A		N/A	[90]
			1:2								
**13**	β-CD:PMDA	Rosuvastatin calcium	1:6	≈82% (2 h, pH = 1.2) 100% (4 h, pH = 6.8)		Not observed	≈65% (2 h, pH = 1.2) 100% (24 h, pH = 6.8)	N/A		N/A	[91]
**16**	β-CD:CDI	Paliperidone	1:4	≈ 45% (3 h) 73.3% (6 h)		Not observed	55.39% (3 h)	N/A		N/A	[94]
			1:8	≈ 50% (3 h) 78.35% (6 h)							
**18**	β-CD:DPC	Camptothecin	1:2	20–25% (24 h) (for all 1:*n*)		Not observed	100% (24 h)	N/A		N/A	[77]
			1:4								
			1:8								
**19**	β-CD:DPC	Clobetasol propionate	1:4	32.39 ± 0.10% (1 h) 55.81 ± 0.60% (6 h) 86.25 ± 0.28% (24 h)		Not observed	73.03 ± 3.84% (1 h) 95.30 ± 3.01% (6 h)	H		Anomalous transport	[96]
**20**	β-CD:DPC	Irbesartan	1:4	(*D:NS* 1:2) 31.55 ± 0.58% (10 min) 91.64 ± 1.47% (2 h)		Not observed	7.05 ± 0.74% (10 min) 35.85 ± 0.346% (2 h)	N/A		N/A	[97]
**21**	β-CD:PMDA	Irbesartan	1:6	(*D:NS* 1:4) 42.35 ± 6.62% (10 min) 72.36 ± 1.49% (2 h)		Not observed	7.05 ± 0.74% (10 min) 35.85 ± 0.346% (2 h)	N/A		N/A	[97]
**22**	2-HP-β-CD:DPC	Ergotamine	N/A	24% (24 h)		Not observed	42% (24 h)	N/A		N/A	[98]
**23**	β-CD:CDI	Atorvastatin calcium	1:4	100% (6 h)		58.1 ± 5.6% (1 h)	43.6 ± 5.3% (6 h)	N/A		N/A	[99]
**25**	β-CD:CDI	Acyclovir	1:4	70% (3 h)		Not observed	N/A	N/A		N/A	[101]
**26**	β-CD:SA	Acyclovir	N/A	22% (3 h)		Not observed	N/A	N/A		N/A	[101]
**28**	2-HP-β-CD:DPC	Ibuprofen	1:4	≈ 94% (2 h)		≈35% (20 min)	≈45% (2 h)	First order		Fickian diffusion	[102]
**31**	β-CD:DPC	Efavirenz	1:4	51.88 ± 0.808% (90 min)		Not observed	27.33 ± 1.22 (90 min)	N/A		N/A	[105]
**32**	β-CD:CDI	Erlotinib	1:4	71.26 ± 0.54% (1 h)		Not observed	36.21 ± 5.09% (1 h)	H		Fickian diffusion	[106]
**34**	β-CD:CDI	Rilpivirine	1:4	56% (1 h)		Not observed	≈30% (1 h)	N/A		N/A	[107]
**35**	β-CD:PMDA	Rilpivirine	1:4	68% (1 h)		Not observed	≈30% (1 h)	N/A	N/A		[107]
**41**	β-CD:PMDA	Paclitaxel	1:4	4% (24 h, pH = 7.4) 10% (24 h, pH = 5.5)		Not observed	N/A	N/A	N/A		[112]
**42**	β-CD:DPC	Dexamethasone	1:2	≈40% (5 h)		Not observed	≈30% (5 h)	N/A	N/A		[113]
			1:4	≈75% (5 h)							
			1:8	≈35% (5 h)							
**43**	β-CD:CDI	Econazole nitrate	1:8	≈ 77% (24 h)		Not observed	≈100% (4 h)	H	Fickian diffusion		[114]
**45**	β-CD:CDI	Paclitaxel	1:4	100% (2 h)		100% (2 h)	N/A	N/A	N/A		[116]
**46**	β-CD:PMDA	Imiquimod	1:4	≈35% (24 h)		Not observed	4% (24 h)	KP	Non-Fickian diffusion		[117]
**47**	β-CD:DPC	Methotrexate	1:4	67.67 ± 0.77% (72 h, pH = 7.4) 63.52 ± 3.41% (72 h, pH = 6.8)		Not observed	95% (4 h, both pH)	N/A	N/A		[118]
**48**	β-CD:DPC	Flurbiprofen	N/A	≈10% (2 h)		Not observed	N/A	N/A	N/A		[45]
**49**	β-CD:DPC	Imatinib mesylate	1:1.5	88.87 ± 2.45% (30 min)		Not observed	98.29 ± 3.65 (20 min)	N/A	N/A		[119]
**50**	β-CD:DPC	Paclitaxel	1:2 1:4 1:8	N/A		Not observed	N/A	N/A	N/A		[120]

Abbreviations: 1:*n*, CD/cross-linker molar ratio; H, Higuchi model; KP, Korsmeyer–Peppas model.

The change in 1:*n* is one of the influential factors on drug release (Table 8). An increase in cumulative drug release with increasing 1:*n* was observed for β-CD:CDI complexes with flutamide (**2**) [80] and piroxicam (**3**) [81], whereas for β-CD:DPC complexes with febuxostat (**6**) [84] it occurred due to an increase of porosity and surface area, enabling the binding of a greater amount of the drug and enhancing its dissolution [81], and a denser polymeric network, leading to the binding of a larger amount of drug in the form of non-inclusion rather than inclusion complexes, due to steric hindrances preventing them from reaching the CD cavities [80,84]. The decrease in the amount of drug released with increasing 1:*n* occurred for β-CD:CDI complexes with bortezomib (**1**) [79], sulfamethoxazole (**4**) [82], and tamoxifen (**30**) [104], as well as for paclitaxel-loaded β-CD:DPC (**50**) [120], most probably due to slower diffusion of the drug through the densely cross-linked polymer network [82,104]. Cumulative drug release was observed for β-CD:DPC complexes with camptothecin (**18**) [77] and dexamethasone (**42**) [113], as well as acetylsalicylic acid-loaded β-CD:PMDA (**11**) [89], and increased with 1:*n* in the manner 1:2 < 1:8 < 1:4, showing that slow drug release from a polymer network that is too loose or too dense occurs mainly due to the formation of inclusion complexes coming from too few drug-binding sites and steric hindrances during drug loading, respectively.

Another important factor of drug release is the cross-linker type, which can affect the drug transport through the CDNS network. A fast release can occur due to the formation of appropriate interactions conducive to drug diffusion, as observed for diclofenac sodium-loaded β-CD:PMDA (**12**) [90], where strongly hydrophilic carboxyl groups of the cross-linker interact with hydrophilic moieties of the drug and, thus, enhance the transport and wettability of the drug. However, the formation of excessively strong interactions between the drug and the cross-linker could be the cause of prolonged release. The comparison of drug release data of acyclovir-loaded β-CD:CDI (**25**) and β-CD:SA (**26**) showed a slower release rate of the latter due to stronger interaction with the drug, thanks to the hydrophilic nature of the cross-linker [101]. Also, the organization of the polymer network affects drug diffusion, as can be seen from the example of crystalline nanosponges. Crystalline dexamethasone-loaded β-CD:DPC (**26**) [100] present a highly organized crystal structure through which diffusion of the drug occurs fluently, whereas paracrystalline CDNSs have an irregular structure, which results in retardation of drug diffusion.

Drug-loaded CDNSs can show potential pH-dependent release properties. The drug release profiles of naproxen-loaded β-CD:TDI (**7**) [85] and β-CD:PMDA complexes with paclitaxel (**41**) [112] and doxorubicin (**51**) [121] showed enhanced release rates in acidic environments. On the other hand, doxorubicin-loaded β-CD:DPC (**52**) [45] showed a more sustained release at acidic pH in comparison with the physiological one. The mechanism of pH dependence is still unknown, but it is certainly related to the durability of different ionic forms of the drug in different pH environments.

Overall, the release rate of nanosponges depends on diffusion of the drug through the CDNS structure and the dissolution properties of the CDNS. Both of these factors can be influenced by changes in nanosponge properties [48]. The basis of diffusional properties consists in the density of the polymer network (1:*n*) and the types of interactions between the drug and CDNS that take place during the diffusion process (mostly dependent on the CD and cross-linker type). Simultaneously, the dissolution properties of CDNSs are strictly related to their microscopic properties, which can be determined using the Noyes–Whitney equation [153]:

(12) $$\frac{dC}{dt}=\frac{DA}{h}\left(C_{s}-C_{x}\right)$$

where dC/dt is the release rate, D is the diffusion coefficient, A is the surface area, h is the diffusional distance (between which the concentration gradient occurs), C_s_ is the saturation solubility, and C_x_ is the bulk concentration.

According to Equation (12), the dissolution rate of the drug can be increased by the higher concentration gradient between the CDNS and the drug release site (related to EE), surface area enlargement, and the decrease in diffusional distance (related to particle size and 1*:n*) [60]. We found that all of these relations had already been observed in previous research, but the precise description and understanding of each characteristic influencing the release profile need further investigation.

For a thorough evaluation of drug release kinetics, the obtained release data are fitted to a variety of kinetics models, where the determinant of the best fit is the highest determination coefficient (R^2^) value [154]. Each model describes different release behavior, dictated primarily by the structure of the polymer [155]. Table 9 presents a collection of frequently used kinetics models.

Zero-, first-, and second-order kinetics models present simple models of drug release that accurately describe the release process only partially, too ordinary for polymer matrices. The zero-order model is concentration-independent—the amount of the released drug is constant and could appear following the initial burst release and describe the controlled release during which the release rate remains constant. The first-order kinetics appears for release from water-soluble porous material, where the release rate is based on the drug concentration gradient, whereas the second-order kinetics best fits poorly soluble substances encapsulated in transport matrices [154,156].

More sophisticated release models have been designed to describe the release subject to a given release mechanism. Fitting the release data into the Korsmeyer–Peppas model provides information about the type of mechanism governing the drug transport during the release [154]. The value of the diffusional exponent *n* in the Korsmeyer–Peppas equation indicates a few possible mechanisms for different three-dimensional models (Table 10) [154].

Polymers change their elastic behavior depending on ambient temperature, the critical value of which is called the glass transition temperature of the polymer (T_g_). At temperatures above *T_g_,* polymer chains are characterized by high mobility, enabling easy penetration of the solvent into the polymer network. This situation is characteristic of *diffusion-controlled drug release*, described by Fickian diffusion (case I transport) and *quasi*-Fickian diffusion models, where the diffusion rate of the solvent R_diff_ is slower than the polymer relaxation rate R_relax_ (R_diff_ << R_relax_). On the other hand, non-Fickian diffusion occurs at temperatures below T_g_, when the low mobility of polymer chains does not allow for prompt penetration of the solvent. Three types of non-Fickian models can be distinguished [73,154,155,156,166,167]:

- Anomalous transport, where drug transport is dependent on both Fickian diffusion and swelling and relaxation of the polymer network; this situation results in equalization of the solvent diffusion rate and polymer relaxation rate (*R_diff_*~*R_relax_*), representing the transition state between diffusion- and swelling-controlled models;
- Case II transport, where drug transport is dependent on swelling and relaxation of the polymer network, while being independent of time (zero-order kinetics); in this model, the solvent diffusion rate is faster than the relaxation rate of the polymer (*R_diff_ >> R_relax_*), showing hindered solvent penetration; both anomalous and case II transport can be referred to as *swelling-controlled drug release* models;
- Super-case II transport, where drug transport is dependent mainly on macromolecular relaxation and erosion of polymer chains (*relaxation-controlled drug release*), resulting in fast solvent diffusion velocity, similar to that observed in the case I transport model.

Taking the above into account, the Korsmeyer–Peppas model [157,158] can be used in situations where the drug release mechanism is unknown, or where more than one mechanism occurs [154,156]. Since Fickian diffusion shows time dependence by the square root of time (*n* ≈ 0.5), the Higuchi model [159] (the progenitor of release profile models [156]) works best as an approximator of drug release via diffusion, where the solvent penetrates inside the CDNS, dissolves the drug, and transports it outside the polymer according to Fick’s law [82,168]. Despite being two of the best approximators of drug release from polymer matrices, the Higuchi and Korsmeyer–Peppas models describe the diffusion-based transport with good approximation only up to a cumulative drug release of 60% (M_t_/M_∞_ = 0.60) [168,169]. Therefore, the Peppas–Sahlin model [170] was developed for better approximation of drug release with ongoing anomalous transport [156]:

(13) $$\frac{M_{t}}{M_{\infty }}=k_{1}t^{m}+k_{2}t^{2m}$$

where m is the Fickian diffusional exponent, dependent on the diameter/thickness ratio of the system. The first term represents the Fickian diffusion contribution, whereas the second term expresses the polymer chain relaxation contribution.

Apart from the above, more advanced release profile models have recently been proposed. The Baker–Lonsdale model [160] expands the Higuchi model to better describe the controlled release from spherical matrices [154,156], as does the Hixson–Crowell model [161]. The latter uses the fact that the surface area of particles is proportional to the cube root of their volume, which decreases during drug release. With reducing volume, the loaded drug amount also decreases; thus, the cube root of the unreleased drug fraction–time relationship is established [168,171]. This phenomenon is used to evaluate the drug release from spherical particles, whose geometric shape diminishes during drug release, limited by the dissolution velocity and not by diffusion [154,156]. The Hopfenberg [162] and Cooney [163] models are good approximators for systems characterized by zero-order kinetics drug release with ongoing erosion (case II transport) in different geometries [154,156]. The Weibull model [164,165] approximates the drug release well for all mechanisms, and it is used for the comparison of drug release profiles between different polymer matrices [156,168]. This model assumes that drug release is based mostly on the drug dissolution process, but it is criticized for not possessing any kinetics basis and not having a connection with the dissolution rate of the material [154].

Of all of the abovementioned kinetic models, the Higuchi and Korsmeyer–Peppas models appeared to be the most common (Table 6)—only the ibuprofen-loaded 2-HP-β-CD:DPC (**28**) [102] release profile showed the best fit to the first-order kinetics model. The Higuchi model was the best-fitting model for the kinetics evaluation of sulfamethoxazole’s (**4**) [82] release from complexes with β-CD:CDI, for all studied 1:*n* values, but also for β-CD:CDI complexes with erlotinib (1:4) (**32**) [106] and econazole nitrate (1:8) (**43**) [114] and β-CD:DPC complexes with rilpivirine (1:4) (**10**) [88] and clobetasol propionate (1:4) (**19**) [96]. On the other hand, the Korsmeyer–Peppas kinetics model showed the best fit to the release data obtained for baricitinib-loaded β-CD:DPC (1:4,5) (**5**) [83], nifedipine-loaded β-CD:DPC (**9**) [87], and imiquimod-loaded β-CD:PMDA (**46**) [117]. Amin et al. [84] working on the drug release kinetics of febuxostat-loaded β-CD:DPC (**6**), showed that the 1:*n* dependence–release profiles of 1:*n* 1:4, 1:6, and 1:8 best fit the Higuchi model, after which a switch to the Korsmeyer–Peppas model occurred for 1:*n* 1:10. Simultaneously, the results obtained by Gaber et al. on the release profiles of piroxicam-loaded β-CD:CDI (**3**) [81] showed indirect dependence of the release profile fit on both 1:*n* and *D:NS*, at times with identical R^2^. On the other hand, R^2^ of the Higuchi kinetics model fitting the release of sulfamethoxazole from β-CD:CDI (**4**) decreased with increasing 1:*n*, where slower drug release is expected due to more intense cross-linking [82]. These results suggest that with the increase in 1:*n* the release kinetics switches from the purely diffusion-based mechanism described by the Higuchi model to more complicated and diverse systems better portrayed by the Korsmeyer–Peppas model, showing the influence of the cross-linking degree on the drug release profile. However, due to the scarce and diverse results, further research should be performed to confirm the above hypothesis.

When it comes to the drug release mechanism, nanosponges might behave differently than the predicted release profile model. For most of the discussed examples—(**4**) [82], (**5**) [83], (**9**) [87], (**28**) [102], (**32**) [106], (**43**) [114], and (**46**) [117]—an accordance between the kinetics model and the drug release mechanism was observed. For the rest—(**3**) [81], (**6**) [84], (**10**) [88], and (**19**) [96]—Fickian and non-Fickian transport was assigned to the Korsmeyer–Peppas and Higuchi kinetics, respectively. These results show that the boundary between those two models is obliterated, which could become an obstacle in defining the drug release model and mechanism. There are several possible solutions to this problem—the development of more sophisticated mathematical models, or more detailed methods of drug release profile measurement, the latter being easier to carry out. Additionally, Fickian diffusion was the dominant mechanism of drug transport for β-CD:CDI-based complexes—(**3**) [81], (**4**) [82], (**32**) [106], and (**43**) [114]—whereas non-Fickian transport (mainly anomalous transport) occurred to drive the drug release from β-CD:DPC-based complexes: (**5**) [83], (**9**) [87], (**10**) [88], and (**19**) [96]. Since the comparison of properties includes different nanosponge types, 1:*n* values, and model drugs, no definitive conclusions can be formulated without further research.

### 3.10. Computational Studies

Since CDNSs’ properties are influenced by a number of variables, from synthesis to drug formulation, obtaining polymers suitable for a given purpose is not an easy task, being very time- and resource-consuming. This leads to the use of computational methods to optimize the selected CDNS characteristics. Using mathematical models, one can predict the structure and properties of CDNSs or explain them at the molecular level by comparison with the experimentally obtained data. Also, computational methods are used in optimization schemes that make it possible to evaluate the relationship between the synthesis parameters and CDNS properties, and to identify the variables whose changes most affect the CDNS properties. This approach saves valuable time, minimizes the number of syntheses and experimental procedures, and can also improve the quality of the product [57].

#### 3.10.1. Molecular Modeling and Molecular Dynamics

Molecular modeling (MM) is used to determine the most energetically and geometrically stable structure of given molecules by energy minimization of three-dimensional systems. MM methods are used to confirm and present the principles of drugs’ binding into CD cavities. Such studies have been performed for different models, e.g., β-CD:DPC complexes with irbesartan (**20**) [97], efavirenz (**31**) [105], and irbesartan-loaded β-CD:PMDA (**21**) [97]. On the other hand, molecular dynamics (MD) uses Newton’s equations of motion to predict the evolution of the system over time at a constant temperature, which is used to study the kinetics of the CDNS structure. Raffaini et al. [172] studied the dynamics of water molecules incorporated in a model of non-loaded β-CD:PMDA, consisting of six β-CD molecules linked with PMDA at a 1:1 molar ratio in a circular manner, with an additional tail of two β-CD moieties. The trajectory of the water molecules during simulation enabled the evaluation of their mean square displacement (MSD), which is correlated with the diffusion coefficient. The results of the simulations confirmed the existence of three water molecule types: a low-mobility type, associated with water creating H-bond networks in CD cavities; a high-mobility type, consisting of free, not strictly bound water molecules in CDNS channels; and a third type, not observed experimentally, probably representing water molecules adhered at the CDNS surface, showing medium mobility. The computationally obtained diffusion coefficients of low- and high-mobility water molecules showed very good agreement with the experimental results obtained by MAS NMR experiments [173], showing high precision of the mathematical models used to describe CDNS dynamics. The same group studied the influence of drug concentration on four models of β-CD:PMDA complexes with piroxicam, showing that at lower concentrations drug molecules are easily incorporated into CD cavities and, to some extent, adhere to the surface of CDNS molecules, whereas at higher concentrations the formation of piroxicam aggregates occurred, disabling the fluent incorporation inside the CDNS and showing the importance of the choice of *D:NS* before drug loading [174].

#### 3.10.2. Structure Optimization Methods

To obtain nanosponges for a given purpose, a series of synthesis and physicochemical studies must be performed for each CDNS individually. Even then, the CDNS might not show the most suitable properties, since the range of synthesis factors like temperature, reaction time, pH, pressure, stirring rate, concentration, etc., is very wide, and the relationships between different factors are hard to evaluate [68,140]. Instead of further synthesis, statistical theoretical methods can be used in this manner as optimization procedures for the CDNS synthesis. Optimization allows one to predict the conditions best fitting the synthesis with the most suitable properties, with a reduced number of experimental runs needing to be performed. Optimization procedures are usually based on response surface methodology (RSM), a group of statistical methods that enable the establishment of the relationships between several independent variables (factor—synthesis conditions) and dependent variables (response—synthesis yield or physicochemical properties of the product). Each factor has an assigned response coefficient, showing its importance in the overall changes in the dependent variable value. After establishing the factors, the design of experiments (DoE) is implemented for planning a series of synthesis routes to evaluate the synthesis–properties relationship. There are three DoEs that are usually selected: full factorial design (FFD), central composite design (CCD), and Box–Behnken design, which generate a series of synthesis conditions, after which the physicochemical properties of the obtained CDNS are used to approximate the response using generated polynomial regression equations that can take linear or polynomial forms.

Each subsequent design becomes more efficient by limiting the number of runs needed for the experiment. Each of the terms of the regression equations is checked for statistical significance (ANOVA) to evaluate which relationships between independent and dependent variables have the biggest impact on the course of synthesis [68,114,140].

The obtained polynomial regression equations are indistinct, so various methods of graphical representation are used. Pareto charts, which present the outcome of each variable and its reasonable significance, are used for graphical visualization of the results [114]. Perturbation plots show how the changes in factor values influence the response value at different levels. Comparison of perturbation plots for all considered factors helps to find the factors most affecting the response. The 2D contour plots and 3D response surface plots show how the response changes in a two-factor grid of factors.

After data analysis, the optimized conditions are implemented into the experiment to synthesize the optimized CDNS, which is examined with a variety of physicochemical methods to confirm the response values obtained via theoretical optimization. Sometimes, additional confirmation experiments are performed with various concentrations of ingredients to confirm the validity of the theoretical model [68,140]. Such experiments, using all three mentioned DoEs, were carried out for β-CD:DPC, the details of which are presented in the following articles [68,119,140].

An important work by Pushpalatha et al. [175] presents the screening process using hierarchy analysis for the best cross-linker type for CDNS preparation. The division criteria included synthesis conditions (method, time, temperature, and the equipment needed for the synthesis), variables referring to the use of a given cross-linker (the structure of the cross-linker, 1:*n*, catalysts, and environmental requirements needed for the synthesis), and characteristics of the obtained CDNS (particle size and surface, crystallinity, and solubility of the obtained CDNS). Each of the criteria was investigated for four cross-linker types: carbonyls, diisocyanates, anhydrides, and epichlorohydrin. A nine-step process was used to evaluate the validity between subcriteria, taking into account the cross-linker type. Carbonyl cross-linkers turned out to be the most suitable for the synthesis of CDNSs as drug transporters, owing to their rigid, crystalline structure, as well as both chemical and thermal stability. The second-best cross-linkers were anhydrides, due to their low thermal stability, caused by their ability to form hydrogels. This property, together with anhydrous conditions needed during the synthesis, made diisocyanates the third-most-suitable cross-linkers. Epichlorohydrin was the least suitable for CDNS synthesis, due to the high dependence of its structural stability on the pH value. Additionally, the most important criteria appeared to be the synthesis conditions, showing how crucial the synthesis process is for the proper formulation of desirable CDNSs.

### 3.11. Other Analytical Methods

Apart from the analytical methods generally used for CDNS studies, there are some methods that are rarely applied, albeit worthy of interest. One of them is field-emission scanning electron microscopy (FESEM), which is probably the most precise imaging method. The successor of SEM was used by Rao et al. [105] to confirm nanochannel formation in DPC-based nanosponges, and by Suvarna et al. [97] to investigate the changes in PMDA- and DPC-based CDNSs’ particle surface morphology occurring due to drug encapsulation. Another imaging method, known as atomic force microscopy (AFM), was used by Swaminathan et al. [100] to measure the crystal size and shape of DPC-based nanosponges.

As we have already mentioned, mass spectrometry (MS) can be used in the evaluation of drug concentration during drug release experiments. However, Yasayan et al. [82] used MALDI-TOF MS to study the correctness of the conduced synthesis by tracking the peaks of CDI-based nanosponges’ ions and their decay products. This approach makes MS yet another method to confirm the CDNS synthesis.

Circular dichroism has never been used directly during the investigation of CDNS complexes, but it was chosen by some authors during experiments performed on CD complexes. This method enables the evaluation of interactions between the encapsulated substance and CD cavities [176]; thus, it could be useful in detailed studies of drug–CDNS complex formation.

So far, the abovementioned methods have not been used frequently, but they possess potential to enter the group of standard physicochemical methods applied in CDNS studies.

## 4. Conclusions

**Figure 10:**
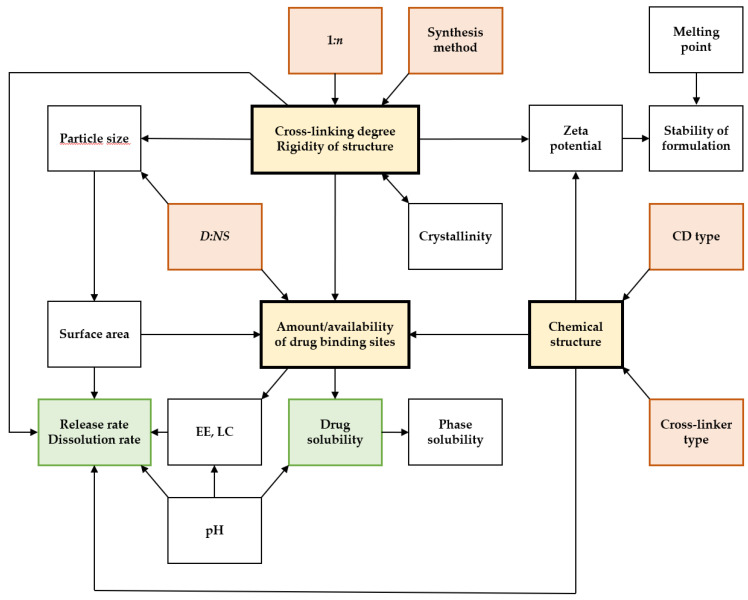
The network of relationships occurring between synthesis conditions and starting parameters (marked in orange) and CDNSs’ physicochemical properties, with particular emphasis on the most influential factors of drug release properties (marked in green). The boxes marked yellow represent important, nodal points of the whole relationship network.

Based on the knowledge of various dependencies between different properties of nanosponges, obtained through the work of numerous research groups, a network of relationships can be established, as presented in Figure 10. However, some of the relationships require further investigation for full confirmation of their existence.

First of all, a group of synthesis parameters (synthesis method, 1:*n*, *D:NS*, CD type, and cross-linker type) can be identified whose changes contribute to the transformation of the CDNS properties. The choice of a suitable synthesis method provides different mixing efficiency of the substrates and a different level of homogenization, translating into changes in the cross-linking process. Fine mixing, achieved via ultrasound-assisted methods, produces CDNSs with denser polymer networks than those synthesized using the melting or solvent methods. This leads to the formation of smaller nanosponge particles with highly organized and rigid crystal structures. Firstly, the decrease in the radius of the particles results in an overall increase in the surface area, providing additional drug-binding sites, mainly at the surface of particles, which leads to increases in the EE and LC. Secondly, crystalline nanosponges possess well-defined and repeatable drug-binding sites in their structure (in nanochannels), which facilitate the transport of drug molecules through the network and to the binding site and influences the drug loading abilities. Any damage to the crystal structure of a CDNS disrupts its properties, leading to a decrease in its loading abilities. However, the transport through less rigid structures is also favorable due to the more fluent polymeric network capable of fitting the transported molecules. Additionally, the ability to form hydrogen bonds with solvent water molecules improves the drug loading capacity. Thus, the influence of the crystallinity of CDNSs is an avenue for further research. Furthermore, the increase in the amount and availability of drug-binding sites connected with the method of synthesis translates into better solubilizing properties, either directly, by control over the rigidity of the structure, or indirectly, by particle size and surface area.

Another critical factor of CDNSs’ properties is their chemical structure, which depends on the applied CD and cross-linker type. The suitable chemical structure affects the EE and LC values by the possibility of formation of favorable interactions between the CDNS and drugs, helping them to reach and bind to the drug-binding sites. Also, CDNSs rich in chemically active cross-linkers create additional binding sites, resulting in better drug-binding abilities, with an additional positive influence on drug solubility. The same phenomenon might affect the electrical mobility of electrons, transferring its influence to zeta potential. This and the abovementioned dependence of zeta potential on 1:*n* are reflected in the stability of the whole formulation, obtained for non-aggregating particles with a relatively high zeta potential. Also, the melting point of the drug–CDNS complex shows how the incorporation of drug molecules into nanosponges protects them against the influence of high temperatures, further contributing to the formulation’s stability.

Another small contributor to CDNSs properties was identified, namely, *D:NS*. Too high a concentration of the drug during loading could result in interactions between drug molecules and even the formation of aggregates of drug molecules, leading to steric hindrances impeding the transport of molecules to binding sites and a decrease in the loading capacity and solubilizing properties of the CDNS. Also, unincorporated drug molecules can adhere to the surface of CDNSs, leading to an increase in particle size.

Apart from the five most important factors, pH was identified as another minor factor influencing CDNSs’ properties, since its effect could be observed during drug loading procedures. The appropriate pH of the solvent could ensure the transformation of the electric nature of the drug to one suitable for the formation of favorable interactions with the CDNS structure, thus facilitating the transport through the polymer network and improving both the drug loading capacity and the drug solubilizing properties.

As described, all synthesis parameters influence the drug’s solubility by various domino-like property relationships. The increase in drug solubility is one of the two most important characteristics of CDNSs, the other being the modifiable release profile (*modifiable* due to various control relationships). The release profile is affected by 1:*n*, while the method of synthesis is affected by the rigidity of the structure, which can restrain the transport of drug molecules due to steric hindrances occurring in highly cross-linked networks, or can facilitate the transport due to loosely linked polymer chains. On the other hand, denser networks possess better developed drug-binding sites compared to uncompleted networks. Also, steric hindrances in densely cross-linked CDNSs entail an increase in the formation of non-inclusion complexes in comparison with inclusion complexes, which might enhance the release of the drug, since the drug molecules are mostly bound in the form of less stable complexes with nanochannels. Furthermore, the 1:*n*, synthesis method, and *D:NS* affect the release properties by their influence on the surface area, but also by the drug loading capacity, both of which have already been described based on the Noyes–Whitney equation. Also, according to that equation, the release rate is influenced by the concentration gradient, which increases with EE (influenced by the 1:*n*, synthesis method, and *D:NS*) and diffusional distance, which is regulated by particle size. Another important influential factor is the chemical structure of the CDNS, which, similar to the drug loading properties, can affect the release via the formation of favorable or unfavorable interactions with the drug during its transport, along with differences in the strength of binding interactions in inclusion and non-inclusion complexes. Apart from that, the release properties can be affected by pH, which is probably related to the durability of the CDNS in different environments and the transformation of drug molecules to ionic forms that are suitable for release, but the precise mechanisms of these phenomena remain unknown.

In summary, we have identified five synthesis parameters that are influential over a series of nanosponge properties, with drug solubility and drug release properties being the two most important ones. The analysis of the relationship network revealed three nodal points—the cross-linking degree/rigidity of the structure, the amount/availability of the drug-binding sites, and the chemical structure of the CDNS, which create the framework of the relationship network and are responsible for the majority of occurring changes in properties. Thus, the key points of the CDNS characteristics network have been established. CDNSs present versatile features for use as drug transport systems. Over the last 25 years, multiple studies have been performed on single CDNS types, and a variety of drugs have been used to help establish the entire network of linked properties. Now, it is time to systematize different types of CDNS to evaluate possible schemes for CDNS design with a convenient release profile, targeted at specific drugs and suitable for various pharmaceutical uses.

## Figures and Tables

**Table 2:** The influence of 1*:n* and *D:NS* on the drug loading properties of different drug–CDNS complexes.

Influential Factor	Influenced Factor	Result	Examples of Drug–CDNS Complexes					
			β-CD:CDI	β-CD:DPC	β-CD:PMDA	β-CD:TDI	2-HP-β-CD:DMC	2-HP-β-CD:DPC
1:*n*	EE	↗	Bortezomib (**1**) Flutamide (**2**) Piroxicam (**3**) Sulfamethoxazole (**4**)	Baricitinib (**5**) Febuxostat (**6**)	-	Naproxen (**7**)	Clotrimazole (**8**)	-
		Max	-	Nifedipine (1:4) (**9**) Rilpivirine (1:4) (**10**)	Acetylsalicylic acid (1:4) (**11**) Diclofenac sodium (1:2) (**12**) Rosuvastatin calcium (1:6) (**13**)	-	-	-
		↘	l-DOPA (**14**)	5-Fluorouracil (**15**)	-	-	-	-
		Ø	Paliperidone (**16**)	-	-	-	-	Ciprofloxacin (**17**)
	LC	↗	Bortezomib (**1**) Flutamide (**2**)	-	-	-	-	-
		Max	-	Rilpivirine (1:4) (**10**) Camptothecin (1:4) (**18**) Clobetasol propionate (1:4) (**19**)	Rosuvastatin calcium (1:6) (**13**)	-	-	-
*D:NS*	EE	↗	-	Irbesartan (**20**)	Rosuvastatin calcium (**13**)	-	Clotrimazole (**8**)	-
		Max	-		Irbesartan (1:2) (**21**)	-	-	-
		Ø	-	-	-	-	-	Ergotamine (**22**)
	LC	Max	Atorvastatin calcium (1:4) (**23**)	-	-	-	-	-
		↘	-	-	Rosuvastatin calcium (**13**)	-	-	-

Abbreviations: ↗, increase in 1:*n*/*D:NS* causes an increase in EE/LC; Max, maximum EE/LC value occurs for a given 1*:n*/*D:NS* ratio; **↘**, increase in 1:*n*/*D:NS* causes a decrease in EE/LC; Ø, EE/LC is not influenced by changes in the 1:*n*/*D:NS* ratio.

**Table 3:** The influence of 1:*n* and *D:NS* on the drug solubility of different drug–CDNS complexes.

Influential Factor	Result	β-CD:CDI	β-CD:DPC	β-CD:PMDA	β-CD:TDI	2-HP-β-CD:DMC	2-HP-β-CD:DPC
1*:n*	↗	Piroxicam (**3**) Sulfamethoxazole (**4**) Paliperidone (**16**)	-	-	Naproxen (**7**)	Clotrimazole (**8**)	-
	Max	Atorvastatin calcium (1:4) (**23**) Tamoxifen (1:4) (**30**)	Clobetasol propionate (1:4) (**19**) Irbesartan (1:4) (**20**) Ibuprofen (1:4) (**27**) Efavirenz (1:4) (**31**)	Irbesartan (1:6) (**21**)	-	-	Ibuprofen (1:4) (**27**)
*D:NS*	Max	Erlotinib (1:4) (**32**)	Rilpivirine (1:4) (**10**) Irbesartan (1:4) (**20**) Griseofulvin (1:1) (**33**)	Irbesartan (1:2) (**21**)	-	-	-

Abbreviations: ↗, increase in 1:*n* causes an increase in solubility; Max, maximum solubility occurs for a given 1:*n*/*D:NS* ratio.

**Table 4:** The influence of 1*:n* on the particle size and zeta potential of drug–CDNS complexes.

Influenced Factor	Result	β-CD:CDI	β-CD:DPC	β-CD:PMDA	β-CD:TDI	2-HP-β-CD:DPC
Particle size	↘	Paliperidone (**16**)	Rilpivirine (**10**)	Acetylsalicylic acid (**11**)	Naproxen (**7**)	-
	Min	-	Ibuprofen (1:4) (**27**)	Diclofenac sodium (1:2) (**12**) Rosuvastatin calcium (1:6) (**13**)	-	Ibuprofen (1:4) (**28**)
	↗	Bortezomib (**1**) Piroxicam (**3**) l-DOPA (**14**)	Baricitinib (**5**) Nifedipine (**9**)	-	-	-
Zeta potential	↗	-	Febuxostat (**6**)	-	Naproxen (**7**)	-
	Max	Paliperidone (1:4) (**16**)	Rilpivirine (1:4) (**10**) Dexamethasone (1:4) (**42**)	Diclofenac sodium (1:2) (**12**)	-	-
	↘	Bortezomib (**1**)	-	-	-	-

Abbreviations: particle size: ↘, increase in 1:*n* causes a decrease in particle size; Min, minimum particle size occurs for a given 1:*n* ratio; ↗, increase in 1*:n* causes an increase in particle size; zeta potential: ↗, increase in 1:*n* causes an increase in the modulus of zeta potential; Max, maximum modulus of zeta potential occurs for a given 1*:n* ratio; ↘, increase in 1*:n* causes a decrease in the modulus of zeta potential.

**Table 5:** Examples of drug–CDNS complexes presenting different properties based on DSC thermograms.

CDNS	Suppression of Endothermic Peak/ Partial Encapsulation	Disappearance of Endothermic Peak/ Full Encapsulation
β-CD:CDI	Paliperidone (**16**) [94] Atorvastatin calcium (**23**) [99] Tamoxifen (**30**) [104] Econazole nitrate (**43**) [114]	Acyclovir (**25**) [101] Erlotinib (**32**) [106] Dexamethasone (**44**) [115] Paclitaxel (**45**) [116]
β-CD:DPC	Rilpivirine (**10**) [88] 5-Fluorouracil (**15**) [93] Norfloxacin (**29**) [103] Efavirenz (**31**) [105] Gabapentin (**37**) [108]	Baricitinib (**5**) [83] Nifedipine (**9**) [87] Dexamethasone (**24**) [100], (**42**) [113] Griseofulvin (**33**) [60] Domperidone (**39**) [110]
β-CD:EDTA	5-Fluorouracil (**36**) [93]	Rosuvastatin calcium (**13**) [91] Meloxicam (**38**) [109] Imiquimod (**46**) [117]
β-CD:PMDA	Acetylsalicylic acid (**11**) [89]	-
β-CD:TDI	-	Naproxen (**7**) [85]
2-HP-β-CD:DPC	Ciprofloxacin (**17**) [95]	Ibuprofen (**28**) [102]

**Table 7:** The influence of incorporation of a drug into a CDNS on the cumulative drug release of different drug–CDNS complexes.

Increase in cumulative drug release	*Improvement in water solubility* β-CD:CDI: flutamide (**2**), atorvastatin calcium (**23**); β-CD:DPC: rilpivirine (**10**); β-CD:PMDA: irbesartan (**21**), rilpivirine (**35**)	*Reduction in drug crystallinity* β-CD:DPC: rilpivirine (**10**) β-CD:PMDA: irbesartan (**21**)	*Reduction in particle size* β-CD:DPC: rilpivirine (**10**), dexamethasone (**24**); β-CD:PMDA: irbesartan (**21**), imiquimod (**44**)	*Increase in surface area* β-CD:DPC: baricitinib (**5**), rilpivirine (**10**)
Decrease in cumulative drug release	*Formation of inclusion complexes* β-CD:CDI: febuxostat (**6**), paliperidone (**16**), econazole nitrate (**43**); β-CD:DPC: clobetasol propionate (**19**), methotrexate (**45**); β-CD:TDI: naproxen (**7**); 2-HP-β-CD:DPC: ergotamine (**22**)			
No influence on cumulative drug release	*Formation of unstable complexes* β-CD:DPC: 5-fluorouracil (**15**); β-CD:EDTA: 5-fluorouracil (**36**)			

**Table 8:** The influence of 1:*n* on the cumulative drug release of different drug–CDNS complexes.

Influential Factor	Result	β-CD:CDI	β-CD:DPC	β-CD:PMDA
1:*n*	↗	Flutamide (**2**) Piroxicam (**3**)	Febuxostat (**6**)	-
	Max	-	Camptothecin (**18**) Dexamethasone (**42**)	-
	↘	Bortezomib (**1**) Sulfamethoxazole (**4**) Tamoxifen (**30**)	-	Paclitaxel (**50**)

Abbreviations: ↗, increase in 1:*n* cause a increase of cumulative drug release; Max, maximum cumulative drug release occurs for a given 1*:n* ratio; ↘, increase in 1:*n* cause a decrease of cumulative drug release.

**Table 9:** The most frequently used kinetics models in drug release profile evaluation.

Kinetics Model	Kinetics Equation	Linear Time–Concentration/Drug Amount Relationship	Ref.
Zero-order kinetics	$[C]=k_{0}t$	$\left[C\right]\;vs\;t$	[154,156]
First-order kinetics	$\ln\frac{{[C}_{0}]}{\left[C\right]}=k_{I}t$	$\ln\left[C\right]\;vs\;t$	[154,156]
Second-order kinetics	$\frac{1}{\left[C\right]}-\frac{1}{\left[C_{0}\right]}=k_{II}t$	$\frac{1}{\left[C\right]}\;vs\;t$	[154,156]
Korsmeyer–Peppas model	$\frac{M_{t}}{M_{\infty }}=k_{KP}t^{n}$	$\log\frac{M_{t}}{M_{\infty }}vs\;\log\;t$	[157,158]
Higuchi model	$\frac{M_{t}}{M_{\infty }}=k_{H}t^{1/2}$	$\frac{M_{t}}{M_{\infty }}vs\;t^{1/2}$	[159]
Baker–Lonsdale model	321−1−MtM∞23−MtM∞=kBLt	$\frac{d\left(\frac{M_{t}}{M_{\infty }}\right)}{dt}vs\;t^{-1/2}$	[160]
Hixson–Crowell model	${{[C}_{0}]}^{1/3}-{\left[C_{r}\right]}^{\frac{1}{3}}=k_{HC}t$	${\left[C_{r}\right]}^{\frac{1}{3}}vs\;t$	[161]
Hopfenberg model	$\frac{M_{t}}{M_{\infty }}=1-{\left[1-\frac{k_{e}t}{C_{0}\alpha_{0}}\right]}^{m}$	$\frac{M_{t}}{M_{\infty }}vs\;t$	[162]
Cooney model	$f=\frac{{\left(D_{0}-2k_{C}t\right)}^{2}+2(D_{0}-2k_{C}t)(L_{0}-2k_{C}t)}{{D_{0}}^{2}+{2D}_{0}L_{0}}$	-	[163]
Weibull model	$M_{t}=M_{\infty }\left[1-e^{-\frac{{\left(t-T\right)}^{b}}{a}}\right]$	$\log\left[-\ln\left(1-\frac{M_{t}}{M_{\infty }}\right)\right]\;vs\;\log\left(t-T\right)$	[164,165]

Abbreviations: C, drug concentration released at time t; C_0_, initial concentration of the drug; C_r_, drug concentration remaining in the CDNS at time t; M_t_, amount of the drug released at time t; M_∞_, total amount of the drug released; M_t_/M_∞_, fraction of the drug released at time t; f, fractional dissolution rate; t, time; k_0_, k_I_, k_II_, k_KP_, k_H_, k_BL_, k_HC_, k_C_, zero-order, first-order, second-order, Korsmeyer–Peppas, Higuchi, Baker–Lonsdale, Hixson–Crowell, and Cooney kinetic constants, respectively; n, diffusional exponent, corresponding to the diffusion mechanism; k_e_, constant for erosion rate; α_0_, initial radius of the polymer; m = 1 (slab), 2 (cylinder), 3 (sphere); D_0_, initial diameter of the cylinder; L_0_, initial length of the cylinder; T, location parameter, representing the lag time of the release process (usually zero); a, scale parameter, representing the timescale of the process; b, form parameter representing the release curve’s character.

**Table 10:** The dependence of the drug transport mechanism during release on the diffusional exponent *n* of the Korsmeyer–Peppas equation, with time dependence assessed for each mechanism.

		*Quasi*-Fickian Diffusion	Fickian Diffusion (Case I Transport)	Non-Fickian Diffusion		
				Anomalous Transport	Case II Transport	Super-Case II Transport
*n*	Planar	*n* ≤ 0.5	0.5	0.5 < *n* < 1.0	1.0	*n* > 1.0
	Cylinder	*n* ≤ 0.45	0.45	0.45 < *n* < 0.89	0.89	*n* > 0.89
	Sphere	*n* ≤ 0.43	0.43	0.43 < *n* < 0.85	0.85	*n* > 0.85
Time dependence (for planar geometry)		t^−0.5^		t*^n^*^−1^	Zero-order dependence	t*^n^*^−1^
